# Microtiter Plate Cultivation Systems Enable Chemically Diverse Metabolic Footprints During Bacterial Natural Product Discovery

**DOI:** 10.1002/bit.29002

**Published:** 2025-04-26

**Authors:** Anton Lindig, Georg Hubmann, Stephan Lütz

**Affiliations:** ^1^ Chair for Bioprocess Engineering, Department of Biochemical and Chemical Engineering TU Dortmund University Dortmund Germany

**Keywords:** bacterial secondary metabolites, cultivation systems, filamentous bacteria, microbioreactor system, microtiter plate cultivation, molecular networking, natural product discovery

## Abstract

Rediscovery of known structures is a frequent problem in screening for bioactive bacterial natural products (NPs). Highly parallelized microtiter plate cultivation systems (MPCS) can improve the chance to discover novel NPs by testing a multitude of cultivation conditions simultaneously. An in‐depth analysis and comparison of cultivation systems for NP discovery, however, has not been carried out so far. We compared the growth and metabolic footprint of four distinct bacterial species in three MPCS, shake flasks, and stirred tank bioreactors (STR). While the big majority of the cultivation systems provided good growth, we found a considerable divergence in secondary metabolite (SM) formation. The SM space was approximated by the appearance of unique mass features (MFs) in the supernatant extracts throughout the cultivation period. Molecular network analysis was applied to visualize the changes from detected MFs at the molecular level. The cultivation systems had a minor impact on the unicellular growing *Bacillus amyloliquefaciens*. This impact was more pronounced for the tested filamentous bacteria, resulting in a diversified metabolic footprint. The maximal overlap of 31% of produced MFs indicates a lack of comparability between the cultivation systems, resulting in different entries of growth phases and the formation of associated SMs. The detected SMs and its derivatives exhibited structural modification depending on the cultivation system. A comparison of *Streptomyces griseochromogenes* NP profile revealed that MPCS yielded less divergent SM formation than shake flasks. Our comprehensive assessment is the first to demonstrate the impact of cultivation systems on the bacterial metabolic footprint, confirming that MPCS provide a robust platform for the parallelization of bacterial cultivations for the discovery of bacterial NPs and accessing the chemical NP space more broadly.

## Graphical abstract

The utilization of microtiter plate cultivation systems in the discovery of natural products expands the secondary metabolic footprint. Microtiter plate cultivation systems, in particular the 48 well flower plate, facilitate the parallelization of bacterial cultivation and offer a greater degree of comparability to stirred tank bioreactors in comparison to shake flasks.

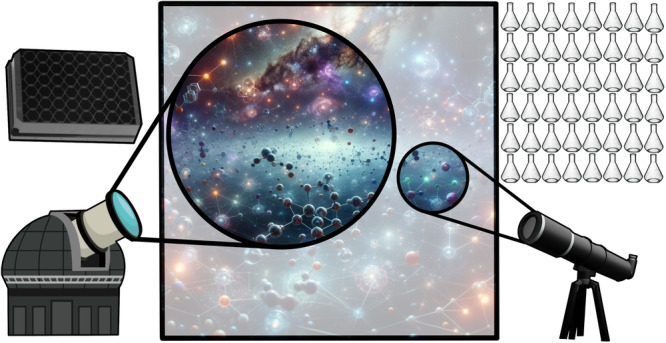

**Abbreviations**
BSF: Baffled shake flask
GYM: Glucose‐yeast‐malt
HPLC: High‐performance liquid chromatography
LC‐MS/MS: Liquid chromatography‐mass spectrometry
maxOTR: Maximum oxygen transfer rate
maxP/V: Maximum volume specific power input
MF: Mass feature
MPCS: Microtiter plate cultivation systems
NP: Natural product
SF: Shake flask
STR: Stirred tank bioreactor
SM: Secondary metabolite
24 DWP: 24‐well deep well plate
96 DWP: 96‐well deep well plate
48 FP: 48‐well flower plate

## 1 Introduction

Bacterial natural products (NPs) remain a valuable source of novel bioactive molecules (Monciardini et al. 2014; Newman and Cragg 2020). The majority of NPs are encoded by dedicated biosynthetic gene clusters and their expression is typically silenced during standard cultivations. Under specific cultivation conditions, the activation of these clusters can be induced. Due to the inherent difficulty in identifying suitable cultivation conditions to induce novel NP production, bacterial NP screening campaigns have frequently resulted in the rediscovery of previously known NPs (Long et al. 2014; Monciardini et al. 2014; Seyedsayamdost 2019). Consequently, an effective method of parallelizing cultivation is required to significantly enhance the throughput in the discovery of novel NPs (Bode et al. 2002; Nothias et al. 2018; Zarins‐Tutt et al. 2016). The use of microtiter plate cultivation systems (MPCS) may prove an effective means of enhancing the throughput for simultaneously testing a multitude of cultivation conditions (Klöckner and Büchs 2012; Lattermann and Büchs 2015).

Cultivation in shake flask (SF) still represents the state of the art in the cultivation of bacteria (Duetz and Witholt 2004; Katz and Baltz 2016; Zarins‐Tutt et al. 2016). In SF cultivations, the maximum oxygen transfer rate (maxOTR) and maximum volume specific power input (maxP/V) reach up to 35 mM/h and 200 W/m^3^, respectively (Table 1). A further increase in both maxOTR and maxP/V is achieved with the use of a baffled shake flask (BSF) (Büchs 2001; Li et al. 2013; Running and Bansal 2016). Over time, a number of different forms of MPCS have been developed and introduced (Long et al. 2014). One variant of MPCS is the unmonitored 24 and 96 deep well plates (DWP) in combination with the Duetz system (Duetz et al. 2000; Duetz 2007; Duetz and Witholt 2004). The cultivation in DWPs results in maxOTR and maxP/V of 38 mM/h and 1000 W/m^3^ for 96 DWP and 110 mM/h and 100 W/m^3^ for 24 DWP (Duetz and Witholt 2004; Running and Bansal 2016; Zhang et al. 2008). Another MPCS is the monitored 48‐well flower plate (FP) with the BioLector system, which permits further monitoring of bioprocess information, including pH and dissolved oxygen (DO) via noninvasive optodes and biomass formation along with fluorescence by back‐scattered light (Funke et al. 2009; Hemmerich et al. 2014; Peter Rohe et al. 2012). The polystyrene microtiter plates contain 48 flower‐shaped deep wells that allow parallel cultivation with maxOTRs of up to 110 mM/h (Funke et al. 2009). The maxP/V of the monitored MPCS has not been determined but is probably ranging between 100 and 1100 W/m^3^. Therefore, all cultivation systems can ensure minimal growth and substrate consumption of microorganisms as a prerequisite for the discovery of novel NPs (Minas et al. 2000; Sohoni et al. 2012; Wang et al. 2017; Zacchetti et al. 2018). A number of studies in the field of NP discovery have already employed with the unmonitored MPCS (Adnani et al. 2015; Duetz et al. 2000; Híreš et al. 2018; Lindig et al. 2023; Minas et al. 2000; Siebenberg et al. 2010; Sohoni et al. 2012) and the monitored ones (Huber et al. 2010; Kensy et al. 2009b; Koepff et al. 2017; Kuhl et al. 2021; Motta Dos Santos et al. 2016; Unthan et al. 2015; Wewetzer et al. 2015).

**Table 1:** Bioprocess parameter of cultivation systems. The various parallel shaken cultivation systems differ in the material, the working volume and the most important bioprocess parameters, that is, the OTR and P/V.

Cultivation systems	Material	Flow pattern	Working volume (mL)	maxOTR (mM/h)	maxP/V (W/m^3^)	Reference
STR	Steel, glass or plastic	defined	> 8	377	1100	(Jossen et al. (2017); Li et al. (2013); Moo‐Young and Butler (2011))
SF	Glass or plastic	defined	> 5	35	200	(Büchs (2001); Li et al. (2013); Running and Bansal (2016))
BSF	Glass or plastic	chaotic	> 5	100	260	(Büchs (2001); Li et al. (2013); Running and Bansal (2016))
48 FP	Polystyrene	chaotic	0.8–1.9	110	—	(Funke et al. (2009))
24 DWP	Polypropylene	defined	0.5–2.5	110	100	(Duetz and Witholt (2004); Running and Bansal (2016); Zhang et al. (2008))
96 DWP	Polypropylene	defined	0.5–1	38	1000	(Duetz and Witholt (2004); Running and Bansal (2016); Zhang et al. (2008))

In liquid cultivation, most bacteria, such as *Bacillales* and *Myxobacteria*, grow disperse in unicellular or in simple multicellular aggregates (Dusenbery 1998; Kumar et al. 2017; Sevugapperumal et al. 2021; Young 2007). The filamentous growing *Actinobacteria* exhibit more complex growth and morphology (Böl et al. 2021) and can form multinucleate hyphae. During submerged cultivation, these hyphae can form pellets, that reach diameters of several millimeters. The bacterial morphology was found to has a profound influence on the NP formation (Minas et al. 2000; Sohoni et al. 2012; Wang et al. 2017; Zacchetti et al. 2018). Only a few studies have described and compared the morphology, growth and the NP formation depending on the cultivation systems (Minas et al. 2000; Siebenberg et al. 2010; Sohoni et al. 2012; Wewetzer et al. 2015). To achieve comparable growth and SM formation in MPCS and SF, the cultivation time was prolonged (Minas et al. 2000), the medium composition and maxOTR were adjusted (Wewetzer et al. 2015) or additives were introduced, including glass beads, 3‐(N‐morpholino)propanesulfonic acid‐buffer (Sohoni et al. 2012) or siloxylated ethylene oxide and propylene oxide particles (Siebenberg et al. 2010). Furthermore, several studies have assessed the scalability from MPCS to stirred tank bioreactors (STR) to establish comparable fermentation courses, growth (Funke et al. 2010; Hemmerich et al. 2014; Kensy et al. 2009a; Koepff et al. 2017; Minas et al. 2000; Sohoni et al. 2012; Unthan et al. 2015; Wewetzer et al. 2015), morphology (Koepff et al. 2017; Sohoni et al. 2012), protein expression (Funke et al. 2010; Hemmerich et al. 2014; Kensy et al. 2009a; Koepff et al. 2017), production of primary metabolites, such as acetate and ethanol, (Wewetzer et al. 2015) and a few selected SMs, including actinorhodin and undecylprodigiosin (Unthan et al. 2015). Nevertheless, it remains unclear whether the entire bacterial SM space and its changes are also dependent on different cultivation systems.

This study is the first to assess the potential of uniquely formed mass features (MFs) as a proxy for the SM space of *Bacillus amyloliquefaciens*, *Corallococcus coralloides*, *Streptomyces griseochromogenes* and *Streptomyces cattleya* during cultivation in SF, unmonitored and monitored MPCS. Additionally, time series analysis of the produced MFs in the cultivation systems was used to evaluate the influence of the cultivation systems on the production profile of SMs over time. The SM space was further decomposed by molecular network analysis to follow the production of identified SMs and their chemical derivatives in the used cultivation systems. The cultivation in MPCS, particularly in 48 FP, resulted in the best performance in terms of growth and total MFs production. Finally, *S. griseochromogenes* was cultivated in bench‐top STR to assess the scalability of bacterial SM profiles obtained in MPCS. Our findings indicate that distinct bacterial strains exhibit varying sensitivities to different cultivation systems, with implications for scalability to STR. This study makes a valuable contribution to the field of parallelized cultivation in bacterial NP screening, providing novel insights for the selection of cultivation systems for future screening campaigns.

## 2 Materials and Methods

### 2.1 Reagents

The chemicals required for the preparation of the culture medium were sourced from Carl Roth GmbH + Co KG (Karlsruhe, Germany). Furthermore, Table S1 provides a comprehensive list of all other chemicals and their respective suppliers.

### 2.2 Strains and Media Composition

The bacteria *B. amyloliquefaciens* DSM 7, *C. coralloides* DSM 2259, *S. griseochromogenes* DSM 40499 and *S. cattleya* DSM 46488 originated from the German Collection of Microorganisms and Cell Cultures (DSMZ, Braunschweig, Germany). The selection of the bacteria strains was based on previous studies, importance as SMs producer and complexity in growth morphology (Schwarz et al. 2021). The growth medium employed was a Glucose‐yeast‐malt (GYM) medium, comprising 4 g/L glucose, 4 g/L yeast extract, 10 g/L malt extract, and adjusted to a pH of 7.2. The used cryo‐cultures in this study contained bacterial broth mixed with 10% (v/v) glycerol, stored at −20°C (Kapoore et al. 2019).

### 2.3 Pre‐Culture of Bacteria

The inoculum cultures of the four bacteria were prepared using 1 mL cryo‐culture and 19 mL GYM medium in 100 mL BSF. The cultures were incubated on an orbital shaker at 200 rpm (50 mm diameter) and 30°C for 24 h. All main cultures in SF, BSF, and MPCS of each bacteria were inoculated from a single pre‐culture. The additional cultivation of *S. griseochromogenes* in STR was inoculated from a separate single pre‐culture.

### 2.4 Cultivation in SF and BSF

For cultivation of SF and BSF, 250 mL flasks were prepared with 2.5 mL of inoculum and 22.5 mL of fresh GYM medium. These flasks were cultivated on an orbital shaker for 5 days at 200 rpm (50 mm diameter) and 30°C. Each bacteria was cultivated in three SF and three BSF. A total of 1 mL of sample was taken from each flask once per day and stored at −20°C. In addition, microscopical pictures were taken from the SF at the end of the cultivation using the Axio Lab A1 (Zeiss, Oberkochen, Germany).

### 2.5 Cultivation in MPCS

For cultivation in 48 FP, each well of the plate contained 0.1 mL inoculum and 0.9 mL fresh GYM medium. The 48 FP was sealed with a gas‐permeable non‐woven sealing foil and was cultivated in a BioLector (Beckman Coulter, Aachen, Germany) for 5 days at 1200 rpm and 30°C. The selected 48 FP in this study contained no optical sensors for the additional detection of pH or DO during cultivation. For the unmonitored MPCS, all wells of the 24 DWP of the Duetz‐system (Adolf Kühner, Basel, Switzerland) contained 0.1 mL inoculum and 0.9 mL fresh GYM medium. The wells of the 96 DWP included 0.05 mL inoculum and 0.45 mL fresh GYM medium. The unmonitored MPCS were closed with a permeable cover plate comprising a stainless‐steel lid, a microfiber filter, a polytetrafluoroethylene filter, and a silicone layer. Both plates were cultivated on an orbital shaker for 5 days at 250 rpm (50 mm diameter) and 30°C. For each bacteria, a separate plate was utilized. In total, triplicates of 1 mL samples were taken once per day and stored at −20°C. For that, three wells per day for 48 FP and 24 DWP were harvested entirely. For 96 DWP, two wells were pooled to obtain 1 mL of culture and three of the pooled wells were totally harvested.

### 2.6 Cultivation in STR

*S. griseochromogenes* was cultivated additionally in the DASbox STR (Eppendorf, Hamburg, Germany) with 20 mL of inoculum and 180 mL of fresh GYM medium. The aeration was set with air to 0.125 vvm and the stirrer speed was kept constant at 1000 rpm. The pH was not controlled throughout the cultivation. In total, 1 mL sample was withdrawn once per day.

### 2.7 Sample Preparation

For analyzing, samples from SF, BSF, MPCS, and STR were thawed at room temperature. All samples were resuspended and transferred completely into a pre‐dried and pre‐weighted microcentrifuge tube. Cells were collected by centrifugation for 20 min at 3795 ×g and 4°C in a SorvallTM Rc 5B Plus centrifuge (Thermo Fisher, Waltham, MA, USA). Supernatant was filtered with a 0.45 μm polyamide filters (Macherey‐Nagel, Düren, Germany) into a glass vial and diluted 1:10 with ultrapure water.

### 2.8 Determination of Growth

The biomass and glucose concentrations were measured for every sample. The biomass concentration was determined by drying all sample tubes with the cell pellet at 80°C until a constant weight was achieved. All tubes were then cooled to room temperature and weighed on a precision scale to obtain the cell dry weight of 1 mL samples. The glucose concentration of all samples was quantified using High‐performance liquid chromatography (HPLC) with a Refractive index detector (Agilent 1200 Series/1260 Infinity, Agilent Technologies, Santa Clara, USA), as previously described in Steinmann et al. 2022. The concentrations were determined from an external calibration curve of standards ranging from 0 to 10 g/L glucose.

### 2.9 LC‐MS/MS Measurement and Data Analysis

The Liquid chromatography‐mass spectrometry (LC‐MS/MS) measurements was performed using an ultra‐HPLC (1290 Infinity II, Agilent, Santa clara, CA, USA) coupled with an electrospray ionization quadrupole time‐of‐flight mass spectrometry (Compact, Bruker, Billerica, MA, USA) with the settings described previously (Lindig et al. 2023). LC‐MS/MS data analysis was conducted using Data Analysis 4.4 (Bruker, Billerica, MA, USA) and MZmine 2.35 (Pluskal et al. 2010). The detailed steps, settings and MFs filtering were applied as described previously (Lindig et al. 2023). Only the MFs which were present in all three samples taken from the cultivation systems were further considered. The MFs from the pre‐culture and the medium control were excluded from all detected masses in the feature lists. The detected MFs were annotated using either chemical standards or library matching with the GNPS online spectral library (Wang et al. 2016) or the software SIRIUS 5.62 (Dührkop et al. 2019), as it has been previously described (Lindig et al. 2023). Examples for annotations are shown in Figures S1, S2.

To assess the production profiles of SMs using time series, we performed hierarchical cluster analysis within the software SPSS (IBM, Ehningen, Germany). For the hierarchical clustering, the ward method was used with an interval measured by squared Euclidean distance and a standardization of −1 to 1 according to the variables. The molecular networks were generated using the default mode of the online GNPS workflow (Wang et al. 2016), as previously described (Lindig et al. 2023). The results of the molecular network analysis were visualized using Cytoscape version 3.6.1 (Shannon et al. 2003). The highest observed abundance of each node in the cultivation systems and labeling of the ions in the molecular network was done as previously described (Lindig et al. 2023). Additionally, the size of the generated pie charts represents the overall highest observed abundance of each node. The production profiles investigated through the time series are displayed in the shape around the pie chart. ChemDraw 20 (PerkinElmer, Waltham, MA, USA) was used to generate chemical structures.

## 3 Results

### 3.1 Growth Characterization in Cultivation Systems

**Figure 1:**
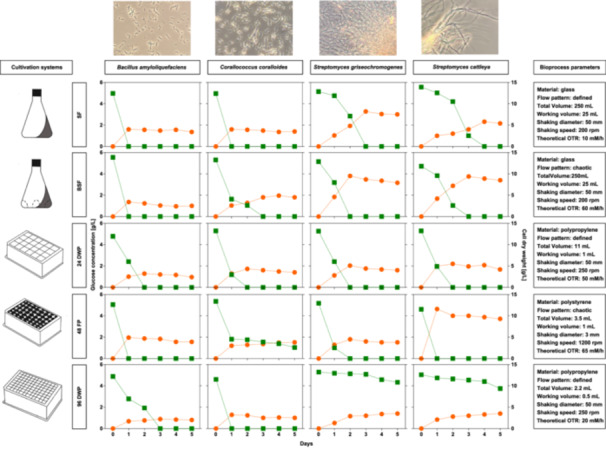
Growth of bacteria in the different cultivation systems. The graph displays the glucose consumption (green squares, left) and cell dry weight (orange circles, right) of *B. amyloliquefaciens*, *C. coralloides*, *S. griseochromogenes* and *S. cattleya* during cultivation in SF, BSF, 24 DWP, 48 FP and 96 DWP. The bacteria were all cultivated in GYM medium for 5 days at 30°C. Differences in bioprocess parameters of the cultivation systems are presented in the boxes on the right. The bioprocess parameters were obtained from literature and manufacturer specifications. Samples were withdrawn each day in triplicates and displayed as means. We calculated a maximum standard deviation of 0.9 g/L for all cell dry weight measurements and 0.3 g/L for all glucose concentrations. Microscopic images of each bacteria were taken at the end of the cultivation from SF.

The growth of the four bacteria, *B. amyloliquefaciens, C. coralloides, S. griseochromogenes* and *S. cattleya*, was characterized over a 5 day cultivation period. All bacteria were cultured in GYM medium at 30°C. Biomass and glucose concentration were measured in triplicates on each day using cell dry weight analysis and HPLC measurements. The Figure 1 displays the biomass formation, glucose consumption, and the process parameters specific to each cultivation system. As expected, we observed a strong inverse correlation between glucose consumption and biomass formation for all tested cultivation systems and bacteria. However, the length of the cultivation to entry into the stationary growth phase, the formed biomass, and the residual glucose differed considerably among the tested cultivation systems. The different cultivation systems had a moderate impact on the length of the growth phases, the biomass formation, and glucose consumption for *B. amyloliquefaciens* (Figure 1). Most of the glucose was consumed within 24 h in SF, BSF and 48 FP, within 2 days in 24 DWP and within 3 days in 96 DWP. Comparable biomass concentrations were obtained in the cultivation systems, with the lowest biomass of 2 g/L recorded in 96 DWP and the highest of 3.9 g/L observed in 48 FP. When cultivating *C. coralloides*, the stationary phase was reached after 1 day when glucose was completely consumed in SF and 96 DWP. The glucose consumption and the biomass formation were slower in 24 DWP and BFS, where the stationary phase was reached at the second day or third day, respectively. Obvious growth differences were observable in the 48 FP, where at the end of the cultivation a residual glucose concentration of 1 g/L was left. This may be attributed to the elevated hydromechanical stress induced by the integrated baffles in the 48 FP, which has been demonstrated to have a negative impact on bacterial growth during the cultivation process (Funke et al. 2009; Khamduang et al. 2009; Toma et al. 1991). The biomass formation varied from a minimum of 2.5 g/L in 96 DWP to maximum of 4.9 g/L in BFS after 5 days of cultivation. Dispersed growth was observed in the different cultivation systems in all cultures of *B. amyloliquefaciens* and *C. coralloides*. The cultivation systems had a pronounced impact on the growth and glucose consumption of the filamentous growing bacteria. In particular, the entry into stationary phase differed in time and the amount of biomass formed in all cultivation systems. In fact, the cultivation in 96 DWP did not reach a stationary phase, as evidenced by the steady increase in biomass formation and the steady decrease in residual glucose over the 5 days cultivation period of all tested filamentous growing bacteria. This may be attributed to the limited oxygen availability in combination with the elevated power input in the 96 DWP (Table 1). This can limit the growth and substrate uptake of the cells, particularly during the cultivation of filamentous *Streptomyces*, which have been described to exhibit sensitivity to variations in mixing and aeration (Minas et al. 2000; Sohoni et al. 2012). Regarding the other cultivation systems, the glucose in *S. griseochromogenes* cultivation was consumed after 3 days in SF and after 2 days in BSF, 24 DWP and 48 FP. The highest biomass concentration of 7.9 g/L was observed in BFS and the lowest of 3.5 g/L in 96 DWP. For *S. cattleya*, glucose was completely consumed within 1 day in 48 FP, 2 days in 24 DWP, 3 days in BSF and within 4 days in SF. At the end of all the cultivations, biomass concentrations ranged between 3.5 g/L in 96 DWP and 9.3 g/L in 48 FP. Additionally, both *Streptomyces* bacteria exhibited heavy pellet formation during cultivation in SF and 96 DWP, with increased growth on the vessel wall (data not shown). Our results show that the growth behavior of the tested bacteria is not only influenced by the oxygen transfer capacity of the different cultivation systems, as indicated by the different theoretical OTRs of the cultivation systems (Figure 1). The shear stress, the used materials and other minor studied factors resulted in a different growth characteristic in the cultivation systems.

### 3.2 Influence of Cultivation Systems on Secondary Metabolome

**Figure 2:**
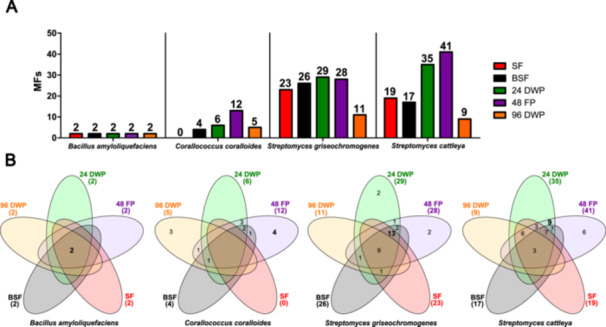
Impact of cultivation systems on metabolic footprint. A The bar chart displays the total number of observed MFs from *B. amyloliquefaciens*, *C. coralloides*, *S. griseochromogenes* and *S. cattleya* during cultivation in SF (red), BSF (black), 24 DWP (green), 48 FP (purple) and 96 DWP (orange). B The Venn diagrams analyze the occurrence of detected MFs. Untargeted metabolomics workflow was used to investigate MFs from the collected supernatant extracts of each sample. These MFs represent detected ions grouped with their observed retention time (rt_*m/z*). Only MFs with an abundance greater than 1000, available MS/MS fragmentation and detection in all triplicates were considered. MFs originating from the pre‐culture and medium control samples were removed.

**Figure 3:**
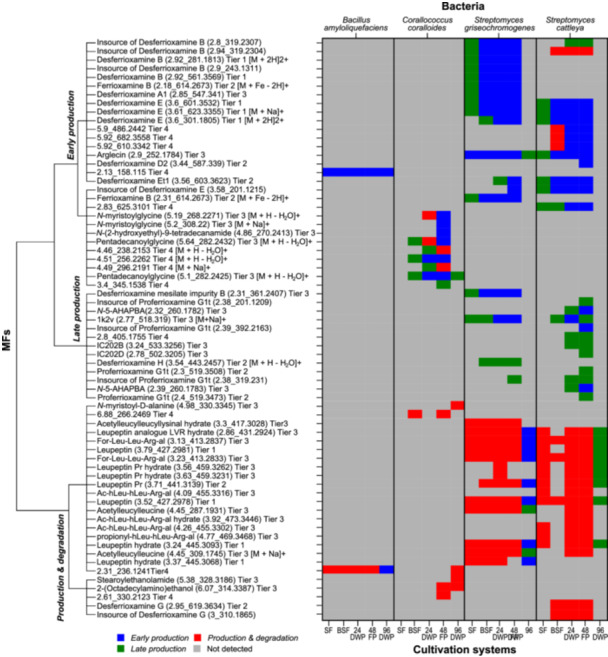
Impact of cultivation systems on SM production profiles during cultivation. Hierarchical clustering analysis of all MFs formed during cultivation of *B. amyloliquefaciens*, *C. coralloides*, *S. griseochromogenes* and *S. cattleya* in SF, BSF, 24 DWP, 48 FP and 96 DWP. The resulting dendrogram displays the clustering of all observed MFs based on their production profiles over time. The heat map displays the production profiles of each MF and bacteria in the different cultivation systems (Figures S4–S6). The profiles are classified as *early production* (blue), *production & degradation* (red), *late production* (green) and no detection (grey). Each MF was labelled with a name, feature (rt_*m/z*) and annotation confidence level (Tier 1 – 4). In‐source fragments are marked with the name of the precursor ion and the feature. All m/z represents [M + H] +, unless specified otherwise.

As a result of the different growth characteristics in the cultivation systems, we studied their impact on the secondary metabolome. We determined the metabolic footprint of the bacteria in the different cultivation systems in an untargeted metabolomics workflow by determining all MFs in triplicate supernatant samples obtained at the end of the cultivation (Figure 2) and over the cultivation period (Figures 3, S3–S6). Only those MFs that were present with an abundance of over 1000 in all triplicates and not detected in the medium control and pre‐culture were further considered.

The MFs obtained for *B. amyloliquefaciens* did not differ among the cultivation systems (Figure 2A), yielding two MFs for all the cultivation systems. When cultivating *C. coralloides*, a maximum of 12 MFs was detected in 48 FP. Of these 12 MFs, only four to six MFs were observed during cultivation in BSF, 24 DWP and 96 DWP. In fact, no MFs were detectable during cultivation of *C. coralloides* in SF. This could be attributed to an optimal growth environment provided by the SF during cultivation of *C. coralloides*, which lacks an additional stressor that is necessary for the activation of the production of SMs (Pan et al. 2019; Romano et al. 2018). The cultivation of *S. griseochromogenes* resulted a maximum of 29 observed MFs in 24 DWP. In the other cultivation systems, MFs ranged between 11 MFs in 96 DWP to 28 MFs in 48 FP. In *S. cattleya* cultivations, a maximum of 41 MFs were detected in 48 FP, followed by 35 MFs in 24 DWP and the other systems with 19 MFs or even less. Next, to investigate the specificity of the detected MFs, we identified unique MFs to each cultivation systems (Figure 2B). No unique MFs were detected during the cultivation of *B. amyloliquefaciens*. In case of *C. coralloides*, four of the twelve MFs were uniquely formed in 48 FP and three additional MFs were found in 96 DWP. In the cultivation of *S. griseochromogenes* and *S. cattleya*, most of the MFs were found to be shared in the different cultivation systems. The highest degree of overlap between the MFs was observed between the 24 DWP and 48 FP for both strains. The differences in MFs appearance across the cultivation systems are likely attributed to the different balance between growth support and additional stress provided by the cultivation systems, as well as the different sensitivity of the bacteria to the specific stressors encountered during cultivation. In conclusion, the metabolic footprints of the bacteria differed clearly between the cultivation systems, although the growth differences appeared to be small.

To further investigate the impact of cultivation systems on the time dependent occurrence of SMs throughout the entire cultivation period, we monitored MF appearance on each day and grouped their intensity profiles over time using hierarchical clustering analysis. In total, the three production profiles *early production*, *production & degradation* and *late production* were classified in this study, with examples provided for each bacterium in Figure S3: The *early production* profile represents MFs that were formed by the bacteria after one or 2 days of growth in the cultivation systems. Their intensity reached a maximum and remained constant until the end of the cultivation. Production profile *production & degradation* was classified when MFs appeared and disappeared during the cultivation. MFs that were produced after 3 or 4 days of cultivation and continuously increased in abundance until the end of cultivation, were categorized as *late production* profile. To gain a deeper understanding of the relation between MFs and their chemical structure, annotations were performed by either comparing retention time and fragmentation spectra to a chemical standard, utilizing the GNPS online spectral library or using the in‐silico fragmentation tool SIRIUS. A comprehensive list of all detected MFs with their annotation is provided in Table S2.

The performed clustering analysis resulted in a dendrogram with two major nodes (Figure 3). The first node contains two sub‐branches with MFs analyzed for their *production & degradation* profile. The second node divides into two branches, one containing MFs classified as *late production* profiles and the other classified as *early production* profiles. The *early production* profiles of MFs are clustered into two additional branches. Based on the clustering of profiles, we analyzed differences in the appearance of SMs over time in the different cultivation systems (Figures 3, S3–S6). During cultivation of *B. amyloliquefaciens*, MF 2.31_256.1241 was observed as an *early production* profile in 96 DWP and appeared to be produced and degraded over time in the other cultivation systems. When cultivating *C. coralloides* in the cultivation systems, differences in the production profile between the cultivation systems were observed. For instance, pentadecanoylglycine exhibited a production profile of *production & degradation* in 24 DWP. In 48 FP, a production profile of *early production* was observed, indicating that no degradation of pentadecanoylglycine had occurred in 48 FP. A *late production* profile was investigated during cultivation in BSF, where it was assumed that an extended cultivation period might be necessary to detect the degradation of pentadecanoylglycine. In general, 48 FP and BSF demonstrated differences in the onset of production of the detected SMs in *C. coralloides*. The same can be observed during the cultivation of the *Streptomyces* bacteria. Overall, the desferrioxamine chemicals exhibited differences in the onset of the production, as exemplified for desferrioxamine E with *late production* in SF and *early production* in the other cultivation systems. The leupeptin molecules showed a lack of degradation, as observed for leupeptin with the production profile of *early* or *late production* in 96 DWP and *production & degradation* in the rest of the cultivation systems. The results of the cluster analysis demonstrate that the tested cultivation systems exerted a pronounced influence on the growth phases of the bacteria and the associated production of SMs. Cultivation systems that exhibited poor growth appeared to lengthen the initial growth phase, thereby reducing the probability of SM production in the presence of a stressor, as most bacterial SMs are produced with the beginning of the stationary phase (Seyedsayamdost 2019). In conclusion, the different cultivation systems clearly influenced the production times and the appearance of SMs in the metabolic footprint of the four bacteria.

### 3.3 Structural Relation and Elucidation of Detected SMs

**Figure 4:**
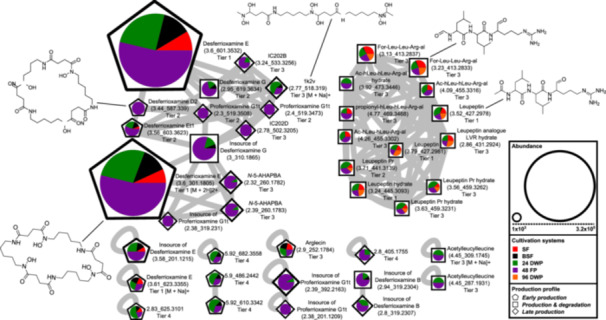
Molecular Network of detected MFs from *Streptomyces cattleya*. The graph displays a molecular network, with the highest detected intensity of each node from *S. cattleya* in SF (red), BSF (black), 24 DWP (green), 48 FP (purple) and 96 DWP (orange), presented in a pie chart. The size of the pie chart displays the overall highest observed intensity in this study. The shape around the pie chart represents the production profiles *early production* (pentagon), *production & degradation* (square) and *late production* (diamond), resulting from the hierarchical clustering analysis of all detected MFs (Figure 3). The nodes represent MFs and are labeled with their chemical structure, name, feature (rt_*m/z*) and the annotation confidence level (Tier 1 – 4). In‐source fragments are marked with the name of the precursor ion and associated MFs. All MFs represent [M + H]+ unless specified otherwise.

To gain further insight into the structural modifications of SMs depending on the cultivation systems, we performed a molecular networking analysis. This allowed for the visualization of the structural relationships between MFs in different cultivation systems (Figures 4, S7–S9). The networks analysis resulted in single and clustered nodes, depending on their fragmentation patterns resulting from the LC‐MS/MS measuring.

For *B. amyloliquefaciens*, we found two single nodes with comparable intensities in all cultivation systems (Figure S7). In case of *C. coralloides*, a molecular family of eight nodes was identified, in addition to seven single nodes, which are variants of *N*‐acyl‐amino‐acids (Figure S8). Four nodes, including *N*‐myristoyl‐*D*‐alanine, stearoylethanolamid and 2‐(Octadecylamino)ethanol, were predominantly produced in 96 DWP, with production profiles over time of *production & degradation* or *late production*. The remaining nodes, including pentadecanoylglycine, *N*‐myristoylglycine and *N*‐2‐(Hydroxyethyl)‐9‐tetradecenamide, were produced primarily in 48 FP, exhibiting all described production profiles over time, with the majority of production occurring in the early phase. For *S. griseochromogenes*, a 10 node family, two double nodes and three single nodes were observed, corresponding to desferrioxamine chemicals (Figure S9). Additionally, one 10 node family and four single nodes, associated with leupeptin molecules, were detected. The desferioxamine chemicals were observed to be mainly produced in 48 FP or equally formed in the cultivation systems, with production profiles over time of *early production* or *late production*. The leupeptin molecules were predominantly produced in 24 DWP or equally detected in the cultivation systems, with production profiles of *production & degradation* or *early production*. Only the precursor of leupeptin, acetylleucylleucine, was detected as primarily produced in 48 FP. In the case of *S. cattleya*, a 14 node family was observed, along with one double node and four single nodes, which corresponded to desferrioxamine chemicals (Figure 4). Additionally, one 13 node family and three single nodes were detected, which were associated with leupeptin molecules. The desferrioxamine chemicals were observed to be predominantly produced in 48 FP, exhibiting all three described production profiles over time. However, the majority of the observed production occurred in the late production phase. Only the derivative 1k2v was detected to primarily produced in 24 DWP. The leupeptin molecules were produced equally in the cultivation systems with the production profiles of *production & degradation*. Only acetylleucylleucine and Ac‐hLeu‐hLeu‐Arg‐al were only detected in 24 DWP and 48 FP. Furthermore, the degradation product of leupeptin, arglecin, exhibited an *early production* profile rather than a *production & degradation* profile compared to the other leupeptin molecules. Additionally, one double node and three single nodes with no annotation were observed to be predominantly produced in 24 DWP during the early production phase, with the exception of 2.8_405.1755, which exhibited the highest production in 48 FP and a *late production* profile over time.

Next, we analyzed the structural modification of SMs and their associated derivatives. During cultivation of *C. coralloides*, differences in chain length and substitution of the amino acids were observed in the formed *N*‐acyl‐amino‐acids (Figure S10). For instance, stearoylethanolamide has an additional propyl group, while *N*‐myristoylglycine misses a methyl group compared to pentadecanoylglycine. 2‐(octadecaylamino) ethanol includes an ethanol moiety and *N*‐myristoyl‐*D*‐alanine an alanine instead of a glycine in pentadecanoylglycine. Additionally, 2‐(octadecaylamino)ethanol is missing one additional oxygen compared to stearoylethanolamide. The *Streptomyces* bacteria produced multiple leupeptin derivatives in this study (Figure S11). The main differences in structural modification of leupeptin chemicals were observed to be substitutions of amino acids and N‐terminal residues. For example, leupeptin analogue LVR contains a valyl instead of a leucyl, acetylleucylleucyllysinal a lysine instead of an arginine and leupeptin Pr an *N*‐propionyl residue instead of *N*‐acetyl compared to leupeptin. In addition to leupeptin chemicals, various desferrioxamine derivatives were produced by the *Streptomyces* bacteria (Table 2). Structural modifications of desferrioxamine included cyclization and differences in chain length. For instance, desferrioxamine E and desferrioxamine Et1, which possess an additional ether structure, represent the cyclic form of desferrioxamine G. Desferrioxamine A1 misses one methyl group, desferrioxamine H is lacking one cadaverine structure and IC202B misses a ketone group in comparison to desferrioxamine B. The analysis on the molecular level may indicate that the production of specific SMs and their derivatives needs specific cultivation conditions, which include a particular combination of stressors derived from the cultivation systems and an optimal balance of stressors and growth support provided by the cultivation systems. The optimal balance appears to differ depending on the complexity of bacterial morphology. This study demonstrates that the different cultivation systems affect the production of different SM profiles and the formation of SM associated derivatives.

**Table 2:** Identified desferrioxamine derivatives in the cultivation systems. List of all identified desferrioxamine structures produced from *S. griseochromogenes* and *S. cattleya* during cultivation in SF, BSF, 24 DWP and 48 FP. The list contains the name, neutral mass, sum formula, detected adducts, annotation confidence level, chemical structure, the bacteria that produced the desferrioxamine and the cultivation systems in which the desferrioxamine were detected.

Name^b^	Neutral mass [g/mol]	Sum formula	Adduct	Tier	Structure	Strain^a^	SF	BSF	24 DWP	48 FP
Des Et1	602.7	C_26_H_46_N_6_O_10_	[M + H] +	2	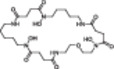	SC, SG	√	√	√	√
Des E	600.7	C_27_H_48_N_6_O_9_	[M + H] +, [M + Na] +, [M + 2H] 2+	1	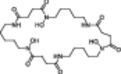	SC, SG	√	√	√	√
Des D2	586.7	C_26_H_46_N_6_O_9_	[M + H] +	2	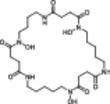	SC				√
Des G	618.7	C_27_H_50_N_6_O_10_	[M + H] +	2	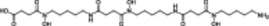	SC		√	√	√
Des B	560.7	C_25_H_48_N_6_O_8_	[M + H] +, [M + 2H] 2 + , [M+ Fe ‐ 2H] +	1	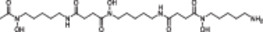	SG	√	√	√	√
Des A1	546.7	C_24_H_46_N_6_O_8_	[M + H] +	3	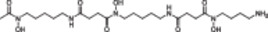	SG	√	√	√	√
IC202B	532.6	C_23_H_44_N_6_O_8_	[M + H] +	3	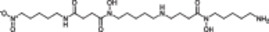	SC			√	√
Pro G1t	518.6	C_23_H_46_N_6_O_7_	[M + H] +	2	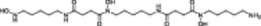	SC				√
IC202D	501.6	C_23_H_43_N_5_O_7_	[M + H] +	3	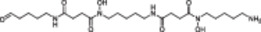	SC				√
1k2v	495.6	C_21_H_45_N_5_O_8_	[M + H] +	3	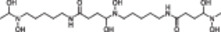	SC		√	√	√
Des H	460.5	C_20_H_36_N_4_O_8_	[M + H ‐ H_2_O] +	2	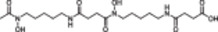	SG		√	√	√
Des mesylate impurity B	360.5	C_16_H_32_N_4_O_5_	[M + H] +	3	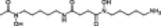	SG	√	√	√	√
*N*‐5‐AHAPBA	259.3	C_11_H_21_N_3_O_4_	[M + H] +	3	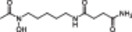	SC			√	√

^a^ SG = Streptomyces griseochromogenes, SC = Streptomyces cattleya.

^b^ Des = Desferrioxamine, Pro = Proferrioxamine, N‐5‐AHAPBA = N'‐[5‐[acetyl(hydroxy)amino]pentyl]butanediamide.

### 3.4 Comparison of Cultivation Systems to STR

**Figure 5:**
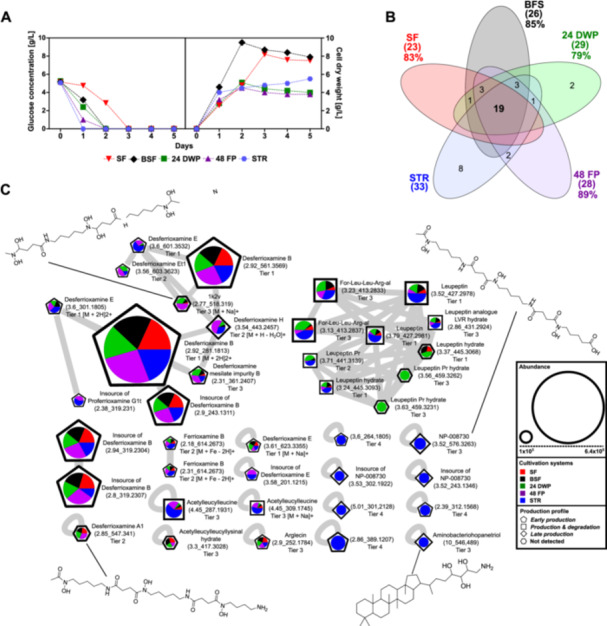
Comparison of growth and SMs space of cultivation systems to STR. A The graph displays the glucose consumption (left) and cell dry weight (right) of *S. griseochromogenes* during cultivation in SF, BSF, 24 DWP, 48 FP and STR. *S. griseochromogenes* was cultivated in GYM medium for five days at 30°C. B The Venn diagram analyze the occurrence of detected MFs and the percentage of shared MFs in each cultivation systems to STR. C The molecular network displays the highest detected intensity of each node in SF (red), BSF (black), 24 DWP (green), 48 FP (purple) and STR (blue), presented as a pie chart. The shape around the pie chart represents the production profiles *early production* (pentagon), *production & degradation* (square), *late production* (diamond) and not detected (hexagon), which were derived from the hierarchical clustering analysis of all detected MFs in STR (Figure S12). The nodes represent MFs and are labelled with their chemical structure, name, the MFs (rt_*m/z*) and the annotation confidence level (Tier 1 – 4). In‐source fragments are indicated by the name of the precursor ion and the associated MFs. All MFs represent [M + H]+ unless otherwise specified.

To determine the degree of similarity of the SM space between the MPCS and STR, we cultured *S. griseochromogenes* in a bench‐top STR over a period of 5 days. The biomass formation, the glucose consumption and the metabolic footprint were measured daily and compared to the other cultivation systems. As one of the most important NP producing species, *S. griseochromogenes* was selected due to the complexity of its growth morphology and its clear response in growth and SMs production to the cultivation systems in this study. The data from the 96 DWP was excluded from the comparison, because *S. griseochromogenes* exhibited poor growth and SM production in this MPCS (Figure 1). In the STR, *S. griseochromogenes* consumed the available glucose within 24 h. This entry point to the stationary growth phase matches well with the growth in BSF, 24 DWP and 48 FP (Figure 5A). The final biomass concentrations obtained in the STR was 5.5 g/L and comparable to the 4 g/L obtained in the MPCS. A total of 33 MFs were detected during the cultivation in STR, which was the highest observed number of MFs of all cultivation systems tested for *S. griseochromogenes* (Figure 5B). These MFs consisted of eight MFs unique to the STR and the others overlapped to a certain degree with the MFs that were already found in the other cultivation systems. The majority of 19 MFs were shared among all cultivation systems. The highest similarity of 89% was found between STR and the 48 FP. The MFs found in the STR were classified in terms of their time‐dependent appearance (Figures S12, S13) and compared to the production profiles observed in the cultivation systems (Figures 3, S5). Overall, the SM production profile observed in STR matches the profiles obtained in the MPCS rather than those obtained in SF and BSF. The molecular network of *S. griseochromogenes* revealed the existence of two 10 node molecular clusters, two double nodes, and seven single nodes related to desferrioxamine chemicals and leupeptin molecules (Figure 5C). All SMs identified in the STR cultivation exhibited comparable intensities to those observed in the cultivation systems, with the exception of leupeptin chemicals and desferrioxamine E, which exhibited the highest observed intensities in STR. As observed during cultivation in the other cultivation systems, leupeptin chemicals were detected during cultivation in STR with a production profile over time of *production & degradation* and desferrioxamine molecules with *early production* or *late production*. Additionally, eight single nodes were identified that were only detected in STR with production profiles of *early production* or *late production*. Three of these single nodes could be annotated to desferrioxamine derivate NP‐008730, which has a carboxyl group instead of an amine group compared to desferrioxamine B. One node of the eight single nodes, was annotated as aminobacteriohopanetriol and belongs to hopanoids, which was hypothesized to play a role in cell biological processes in a variety of bacteria (Welander et al. 2012). The highest degree of comparability of growth and SMs production during the cultivation of *S. griseochromogenes* in STR was observed with the 48 FP, which may be attributed to the highest similarity of oxygen availability, mixing, and power input compared to the other cultivation systems (Table 1) (Koepff et al. 2017). However, the results indicate that during cultivation in STR, other specific combinations of stressors might be generated compared to the other cultivation systems, resulting in specific and unique MFs. In conclusion, our assessment revealed a high degree of similarity in growth and metabolic footprint between MPCS and the STR, thereby increasing the probability of re‐finding the MFs of initial screening in MPCS.

## 4 Discussion

This study demonstrates that there is a clear influence of the cultivation systems on the growth and the metabolic footprint of *B. amyloliquefaciens, C. coralloides, S. griseochromogenes and S. cattleya*. The use of MPCS improves the discovery of bacterial NPs not only due to their capacity to parallelize cultivations but also due to their superior performance in comparison to SF and BSF. The results demonstrate that MPCS, particularly the 48 FP, facilitate enhanced growth, SMs production and result in a greater degree of similarity to STR compared to SF and BSF. In general, the growth, the total number of SMs and their production profiles over time were affected by the different cultivation systems. This could be attributed to three factors: Firstly, the bioprocess parameters, including the material, the OTR and the P/V, of the cultivation systems can greatly impact the growth and the production of bacterial SMs. These bioprocess parameters can act under suboptimal conditions as a source of stress, which are required to activate silent biosynthetic gene clusters and to trigger the production of SMs (Pan et al. 2019; Romano et al. 2018). For example, this would suggest that the cultivated bacteria experienced a higher degree of stress or a specific combination of stressors in the 48 FP compared to the other cultivation systems. In fact, the flower shape of the 48 FP acts as baffles, increasing hydromechanical stress and inhibiting bacterial agglomeration (Funke et al. 2009). Moreover, previous studies have demonstrated that the addition of polystyrene particles and increased oxygen concentrations during cultivation can result to the aggregation of intracellular reactive oxygen species (Baez and Shiloach 2014; Kim et al. 2022). Consequently, the increased level of stress or the specific combination of diverse stressors resulted in a more diverse SM profile during cultivation in the 48 FP. Secondly, suboptimal conditions and the stress experienced during cultivation can have a profound impact on the growth of the bacteria, resulting in different entries into the stationary growth phase or even the absence. This, however, affects the production of SMs, as most bacterial SMs are produced with the beginning of the stationary phase (Seyedsayamdost 2019). Increasing the stationary phase allows the cells, in the presence of a stressor, to modify their SMs or the entire biosynthetic machinery to suit their needs and changes during cultivation (Barona‐Gómez et al. 2004; Kraboun et al. 2023; Pierwola et al. 2004; Senges et al. 2018; Seyedsayamdost 2019). Consequently, the cultivation systems should provide an optimal balance between enabling rapid growth phase to facilitate prolonged stationary phase and an adequate level of stress to stimulate SM production, thereby enhancing the diversity of produced SMs. Lastly, the morphological behavior of the cultivated bacteria in the cultivation systems differs to a large extend. While no influence was observed during cultivation of *B. amyloliquefaciens* and *C. coralloides*, the *Streptomyces* bacteria showed different morphological behavior in the cultivation systems. Cultivating bacteria with complex growth, such as *Streptomyces*, was described to be challenging due to the sensitivity of the cell‐constructs to cultivation parameters, while unicellular growing bacteria remain easier to cultivate and be less sensitive to changes during cultivation (Dusenbery 1998; Minas et al. 2000; Sohoni et al. 2012; Wang et al. 2017; Young 2007; Zacchetti et al. 2018). Additionally, a direct link between pellet morphology of *Streptomyces* bacteria on the production of NPs was described in literature (Minas et al. 2000; Sohoni et al. 2012; Wang et al. 2017; Zacchetti et al. 2018). Therefore, the effect of the cultivation systems on growth and SM production increases with the complexity in cell growth and morphology of the tested bacteria.

The bacteria produced different mixtures and modifications of SMs in response to the cultivation systems. Unique SMs, which were only detected in one cultivation system, exhibited changes in chain lengths, amino acid substitutions or degradation of chemical groups. Any alteration in molecular structure can significantly affect the purpose and bioactivity of a SM. For example, the antibiotic active desferrioxamine A1 just misses one methyl group and the immunosuppressive active desferrioxamine derivative IC202D misses one acetamide compared to the non‐active desferrioxamine B (Chaimaa Katif et al. 2021; Iijima et al. 1999; Iijima et al. 2000). In general, it is suggested that the bacteria modify their SMs to adapt to their current environmental conditions (Spasojević et al. 1999). Therefore, the bacteria shifted their production machinery to form additional SM derivatives with different activities and mixtures to cope with the stress input coming from the cultivation systems.

This study demonstrates that bacterial cultivation in MPCS leads to diverse mixtures of SMs, including representatives of important NP classes such as non‐ribosomal peptides, siderophores, and *N*‐acyl‐amino‐acids. To further increase the SM profiles and activate silent BGCs, additional stress input needs to be applied through methods such as OSMAC (Bode et al. 2002; Pan et al. 2019; Romano et al. 2018). The use of MPCS allows to test multiple conditions simultaneously, enabling the application of more complex methods such as bivariate OSMAC and high throughput elicitor screenings (Lindig et al. 2023; Seyedsayamdost 2014). This significantly increases the number of produced SMs and the likelihood of discovering new NPs. In addition to activating silent BGCs, the purification of the formed SMs is crucial in NP discovery. In most studies, samples are concentrated during sample preparation using purification methods such as liquid extraction (Schwarz et al. 2021; Senges et al. 2018). This ensures the detection of SMs with low concentrations and increases the total amount. Therefore, it is necessary to use a simple and fast purification process to handle an increased throughput of samples when using MPCS. The usage of multi‐well purification plates integrated into an automated high‐throughput purification process can further decrease the workload and increase the amount of SMs (Badawy et al. 2022; Ginsburg‐Moraff et al. 2022). Furthermore, we demonstrated comparable growth and SMs production in STR and MPCS. This will facilitate the development of the fermentation process and scale‐up to supply larger amounts during novel NP discovery, especially when monitorable MPCS such as 48 FP with pH and DO control are implemented in an automated environment. The implementation of automation in the testing of elicitors during the cultivation of bacteria in MPCS has already been achieved through the use of high throughput elicitor screening methods (Seyedsayamdost 2019). Nevertheless, automated testing of different cultivation conditions in MPCS is still in its early stages and the successful scalability of specific cultivation conditions has not yet been fully explored. Hence, further research and development is required to implement a robust automation method for testing multiple cultivation conditions in MPCS, which may become the new challenge in future NP discovery.

## Author Contributions

**Anton Lindig:** conceptualization, methodology, software, validation, formal analysis, investigation, data curation, writing – original draft preparation, visualization. **Georg Hubmann:** conceptualization, methodology, writing – review and editing, supervision. **Stephan Lütz:** conceptualization; resources, writing – review and editing, supervision, project administration.

## Ethics Statement

The authors have nothing to report.

## Conflicts of Interest

The authors declare no conflicts of interest.

## Supporting information

Supporting information_Cultivation systems_Resubmission_New.

## Data Availability

Upon request, research data will be made available. Additional supporting information can be found online in the supporting information section at the end of this article.
