# Supplementary material for: Microtiter Plate Cultivation Systems Enable Chemically Diverse Metabolic Footprints During Bacterial Natural Product Discovery
Source: Biotechnol Bioeng. 2025 Apr 26;122(8):2021–36. doi: 10.1002/bit.29002 (PMC12235219; doi:10.1002/bit.29002)
Supplement: Supplementary file 1 — Supporting information_Cultivation systems_Resubmission_New. [file BIT-122-2021-s001.DOCX]

**Supporting Information**

**Microtiter plate cultivation systems enable chemically diverse metabolic footprints during bacterial natural product discovery**

Anton Lindig ^1^, Georg Hubmann ^1^ and Stephan Lütz ^1*^

1 Chair for Bioprocess Engineering, Department of Biochemical and Chemical Engineering, TU Dortmund University, Emil-Figge-Straße 66, 44227 Dortmund, Germany. Tel: +49 231 755-7488 Email: [anton.lindig@tu-dortmund.de](mailto:anton.lindig@tu-dortmund.de), [georg.hubmann@tu-dortmund.de](mailto:georg.hubmann@tu-dortmund.de) and [stephan.luetz@tu-dortmund.de](mailto:stephan.luetz@tu-dortmund.de)

* Correspondence: Stephan Lütz, Chair for Bioprocess Engineering, Department of Biochemical and Chemical Engineering, TU Dortmund University, Emil-Figge-Straße 66, 44227 Dortmund, Germany. Tel: +49 231 755-7488 Email: [stephan.luetz@tu-dortmund.de](mailto:stephan.luetz@tu-dortmund.de)

**Table of contents**

**Table S1**……………………………………………………………………………….…**Page 3**

**Table S2**……………………………………………………………………………**Page 4 - 11**

**Figure S1**……………………………………………………………………………….**Page 12**

**Figure S2**……………………………………………………………………………….**Page 13**

**Figure S3**……………………………………………………………………………….**Page 14**

**Figure S4**……………………………………………………………………………….**Page 15**

**Figure S5**……………………………………………………………………………….**Page 16**

**Figure S6**……………………………………………………………………………….**Page 17**

**Figure S7**……………………………………………………………………………….**Page 18**

**Figure S8**……………………………………………………………………………….**Page 19**

**Figure S9**……………………………………………………………………………….**Page 20**

**Figure S10**……………………………………………………………………………...**Page 21**

**Figure S11**………………………………………………………………………………**Page 22**

**Figure S12**……………………………………………………………………………...**Page 23**

**Figure S13**……………………………………………………………………………...**Page 24**

**Table S1.** Commercial origin of standards for Tier 1 MFs annotation.

| **Compound name** | **CAS** | **Catalogue** | **Supplier** |
| --- | --- | --- | --- |
| Desferrioxamine B | 70-51-9 | Y0001937 | Sigma |
| Desferrioxamine E | 26605-16-3 | AG-CN2-0150-M001 | AdipoGen |
| Leupeptin | 24125-16-4 | sc-215242A | Santa Cruz Biotechnology |

**Table S2.** List of all detected MFs with the name, neutral mass, sum formula, adduct, maximum abundance, tier level, strain, and Pubchem CID.

| **MF (*rt_m/z*)** | **Name** | **Neutral mass [g/mol]** | **Sum formula** | **Adduct** | **Max abundance** | **Tier** | **Strain**^†^ | **PubChem CID** |
| --- | --- | --- | --- | --- | --- | --- | --- | --- |
| 2.13_158.115 | - | - | - | [M + H] + | 3770 | 4 | BA | - |
| 2.18_614.2673 | Ferrioxamine B | 616.5 | C25H48FeN6O8 | [M + Fe - 2H] + | 3112 | 2 | SG | 46173771 |
| 2.3_519.3508 | Proferrioxamine G1t | 518.6 | C23H46N6O7 | [M + H] + | 1995 | 2 | SC | 88671570 |
| 2.31_236.1241 | - | - | - | [M + H] + | 7725 | 4 | BA, CC | - |
| 2.31_361.2407 | Desferrioxamine mesilate impurity B | 360.5 | C16H32N4O5 | [M + H] + | 48341 | 3 | SG | 20759763 |
| 2.31_614.2673 | Ferrioxamine B | 616.5 | C25H48FeN6O8 | [M + Fe - 2H] + | 3112 | 2 | SG | 46173771 |
| 2.32_260.1782 | *N*-5-AHAPBA^‡^ | 259.3 | C11H21N3O4 | [M + H] + | 14329 | 3 | SC | 145405924 |
| 2.38_201.1209 | Insource of Proferrioxamine G1t | - | - | - | 1621 | - | SC | - |
| 2.38_319.231 | Insource of Proferrioxamine G1t | - | - | - | 1604 | - | SG, SC | - |
| 2.39_260.1783 | *N*-5-AHAPBA^‡^ | 259.3 | C11H21N3O4 | [M + H] + | 14329 | 3 | SC | 145405924 |
| 2.39_312.1568 | - | - | - | [M + H] + | 11760 | 4 | SG | - |
| 2.39_392.2163 | Insource of Proferrioxamine G1t | - | - | - | 28687 | - | SC | - |
| 2.4_519.3473 | Proferrioxamine G1t | 518.6 | C23H46N6O7 | [M + H] + | 1995 | 2 | SC | 88671570 |
| 2.61_330.2123 | - | - | - | [M + H] + | 2416 | 4 | CC | - |
| 2.77_518.319 | 1k2v | 495.6 | C21H45N5O8 | [M + Na ] + | 25253 | 3 | SG, SC | 23585928 |
| 2.78_502.3205 | IC202D | 501.6 | C23H43N5O7 | [M + H] + | 6302 | 3 | SC | 10577363 |
| 2.8_319.2307 | Insource of Desferrioxamine B | - | - | - | 247789 | - | SG, SC | - |
| 2.8_405.1755 | - | - | - | [M + H] + | 4945 | 4 | SC | - |
| 2.83_625.3101 | - | - | - | [M + H] + | 8932 | 4 | SC | - |
| 2.85_547.341 | Desferrioxamine A1 | 546.7 | C24H46N6O8 | [M + H] + | 21294 | 3 | SG | 154585411 |
| 2.86_389.1207 | - | - | - | [M + H] + | 56839 | 4 | SG | - |
| 2.86_431.2924 | Leupeptin analogue LVR hydrate | 430.2 | C19H36N6O5 | [M + H] + | 9567 | 3 | SG, SC | 44267534 |
| 2.9_243.1311 | - | - | - | - | 233593 | 4 | SG | - |
| 2.9_252.1784 | Arglecin | 251.3 | C12H21N5O | [M + H] + | 14460 | 3 | SG, SC | 193170 |
| 2.92_281.1813 | Desferrioxamine B | 560.7 | C25H48N6O8 | [M + 2H] 2+ | 648727 | 1 | SG | 2973 |
| 2.92_561.3569 | Desferrioxamine B | 560.7 | C25H48N6O8 | [M + H] + | 329410 | 1 | SG | 2973 |
| 2.94_319.2304 | Insource of Desferrioxamine B | - | - | - | 247789 | - | SG, SC | - |
| 2.95_619.3634 | Desferrioxamine G | 618.7 | C27H50N6O10 | [M + H] + | 21856 | 2 | SC | 11444934 |
| 3.13_413.2837 | For-Leu-Leu-Arg-al | 412.5 | C19H36N6O4 | [M + H] + | 92237 | 3 | SG, SC | 10001732 |
| 3.23_413.2833 | For-Leu-Leu-Arg-al | 412.5 | C19H36N6O4 | [M + H] + | 92237 | 3 | SG, SC | 10001732 |
| 3.24_445.3093 | Leupeptin hydrate | 444.3 | C20H38N6O5 | [M + H] + | 14642 | 1 | SG, SC | 72429 |
| 3.24_533.3256 | IC202B | 532.6 | C23H44N6O8 | [M + H] + | 4933 | 3 | SG, SC | 9828474 |
| 3.3_417.3028 | Acetylleucylleucyllysinal hydrate | 416.3 | C20H38N4O5 | [M + H] + | 1901 | 3 | SG | 44267475 |
| 3.37_445.3068 | Leupeptin hydrate | 444.3 | C20H38N6O5 | [M + H] + | 14642 | 1 | SG | 72429 |
| 3.4_345.1538 | - | - | - | [M + H] + | 6163 | 4 | CC | - |
| 3.44_587.339 | Desferrioxamine D2 | 586.7 | C26H46N6O9 | [M + H] + | 11992 | 2 | SC | 14671620 |
| 3.52_243.1346 | Insource von NP-008730 |  |  | [M + H] + | 9830 | - | SG | - |
| 3.52_427.2978 | Leupeptin | 426.6 | C20H38N6O4 | [M + H] + | 36459 | 1 | SG, SC | 72429 |
| 3.52_576.3263 | NP-008730 | 575.7 | C25H45N5O10 | [M + H] + | 26727 | 3 | SG | 14459425 |
| 3.53_302.1922 | Insource von NP-008730 | - | - | [M + H] + | 6244 | - | SG | - |
| 3.54_443.2457 | Desferrioxamine H | 460.5 | C20H36N4O8 | [M + H - H2O]+ | 50043 | 2 | SG | 57509383 |
| 3.56_459.3262 | Leupeptin Pr hydrate | 458.3 | C21H40N6O5 | [M + H] + | 4781 | 3 | SG, SC | 13209091 |
| 3.56_603.3623 | Desferrioxamine Et1 | 602.7 | C26H46N6O10 | [M + H] + | 20752 | 2 | SG, SC | 14671623 |
| 3.58_201.1215 | Insource of Desferrioxamine E | - | - | - | 38016 | - | SG, SC | - |
| 3.6_264.1805 | - | - | - | [M + H] + | 1297 | 4 | SG | - |
| 3.6_301.1805 | Desferrioxamine E | 600.7 | C27H48N6O9 | [M + 2H] 2+ | 277830 | 1 | SG, SC | 161532 |
| 3.6_601.3532 | Desferrioxamine E | 600.7 | C27H48N6O9 | [M + H] + | 315471 | 1 | SG, SC | 161532 |
| 3.61_623.3355 | Desferrioxamine E | 600.7 | C27H48N6O9 | [M + Na ] + | 12847 | 1 | SG, SC | 161532 |
| 3.63_459.3231 | Leupeptin Pr hydrate | 458.3 | C21H40N6O5 | [M + H] + | 4781 | 3 | SG, SC | 13209091 |
| 3.71_441.3139 | Leupeptin Pr | 440.6 | C21H40N6O4 | [M + H] + | 12777 | 2 | SG, SC | 13209091 |
| 3.79_427.2981 | Leupeptin | 426.6 | C20H38N6O4 | [M + H] + | 15376 | 1 | SG, SC | 72429 |
| 3.92_473.3446 | Ac-hLeu-hLeu-Arg-al hydrate | 472.3 | C22H42N6O4 | [M + H] + | 1571 | 3 | SC | 124146737 |
| 3_310.1865 | Insource of Desferrioxamine G | - | - | - | 65022 | - | SC | - |
| 4.09_455.3316 | Ac-hLeu-hLeu-Arg-al | 454.6 | C22H42N6O4 | [M + H] + | 2619 | 3 | SC | 124146737 |
| 4.26_455.3302 | Ac-hLeu-hLeu-Arg-al | 454.6 | C22H42N6O4 | [M + H] + | 2492 | 3 | SC | 124146737 |
| 4.45_287.1931 | Acetylleucylleucine | 286.4 | C14H26N2O4 | [M + H] + | 29035 | 3 | SG, SC | 10968127 |
| 4.45_309.1745 | Acetylleucylleucine | 286.4 | C14H26N2O4 | [M + Na ] + | 8271 | 3 | SG, SC | 10968127 |
| 4.46_238.2153 | - | - | - | [M + H - H2O]+ | 1166 | 4 | CC | - |
| 4.49_296.2191 | - | - | - | [M + Na ] + | 1370 | 4 | CC | - |
| 4.51_256.2262 | - | - | - | [M + H - H2O]+ | 4110 | 4 | CC | - |
| 4.77_469.3468 | propionyl-hLeu-hLeu-Arg-al | 468.3 | C23H44N6O4 | [M + H] + | 3294 | 3 | SC | 124146737 |
| 4.86_270.2413 | N-(2-Hydroxyethyl)-9-tetradecenamide | 269.4 | C16H31NO2 | [M + H] + | 2610 | 3 | CC | 87767144 |
| 4.98_330.3345 | N-myristoyl-D-alanine | 299.4 | C17H33NO3 | [M + H] + | 8836 | 3 | CC | 54463945 |
| 5.01_301.2128 | - | - | - | [M + H] + | 6244 | 4 | SG | - |
| 5.1_282.2425 | Pentadecanoylglycine | 299.4 | C17H33NO3 | [M + H - H2O]+ | 3390 | 3 | CC | 53481663 |
| 5.19_268.2271 | N-Myristoylglycine | 285.4 | C16H31NO3 | [M + H - H2O]+ | 2223 | 3 | CC | 72348 |
| 5.2_308.22 | - | - | - | [M + Na ] + | 1628 | 4 | CC | - |
| 5.38_328.3186 | Stearoylethanolamide | 327.5 | C20H41NO2 | [M + H] + | 16164 | 3 | CC | 27902 |
| 5.64_282.2432 | Pentadecanoylglycine | 299.4 | C17H33NO3 | [M + H - H2O]+ | 2567 | 3 | CC | 53481663 |
| 5.9_486.2442 | - | - | - | [M + H] + | 9130 | 4 | SC | - |
| 5.92_610.3342 | - | - | - | [M + H] + | 14720 | 4 | SC | - |
| 5.92_682.3558 | - | - | - | [M + H] + | 4985 | 4 | SC | - |
| 6.07_314.3387 | 2-(Octadecylamino)ethanol | 313.6 | C20H43NO | [M + H] + | 13542 | 3 | CC | 3015553 |
| 6.88_266.2469 | - | - | - | [M + H] + | 9391 | 4 | CC | - |
| 10.03_546.4887 | Aminobacteriohopanetriol | 545.9 | C35H63NO3 | [M + H] + | 6157 | 3 | SG | 11813582 |

^†^*Bacillus amyloliquefaciens* *= BA, Corallococcus coralloides = CC, Streptomyces griseochromogenes = SG, Streptomyces cattleya = SC,* ^‡^*N*'‑[5‑[acetyl(hydroxy)amino]pentyl]butanediamide = *N*-5-AHAPBA


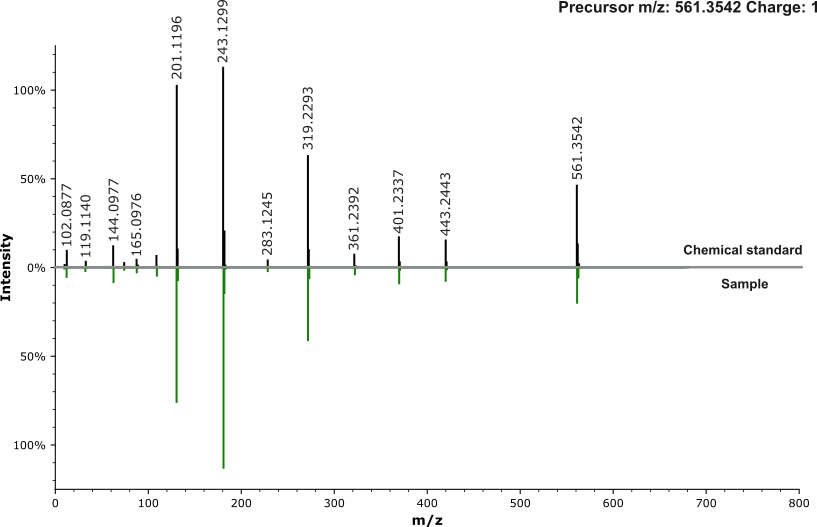


**Figure S1.** Chemical standard annotation for Desferrioxamine B. On top the MS/MS fragmentation pattern from the chemical standard and on the bottom the MS/MS acquired in this study.


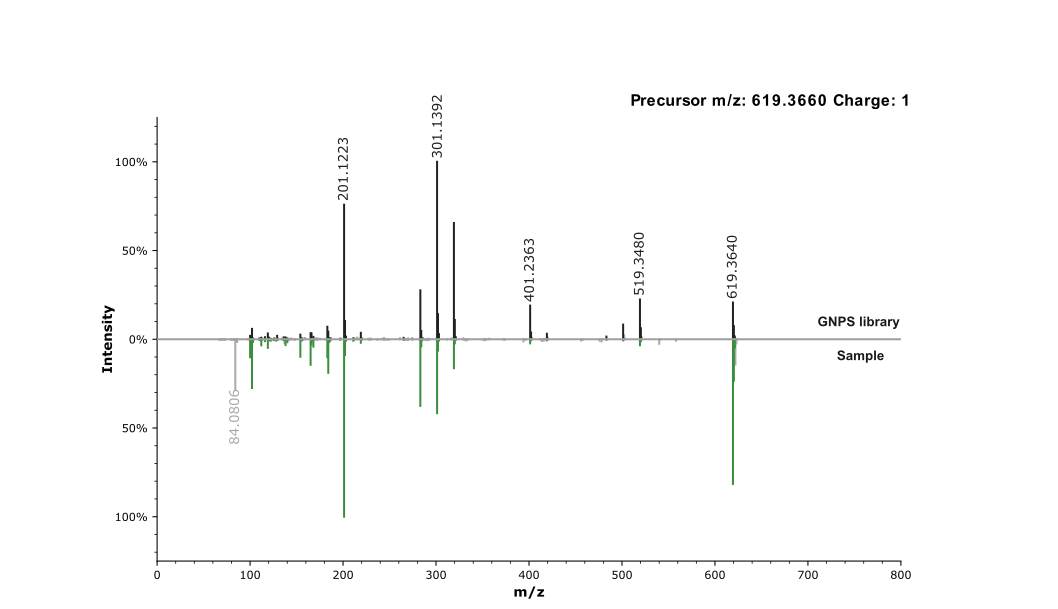


**Figure S2.** GNPS spectral library hit for Desferrioxamine G. On top the MS/MS fragmentation pattern from the spectral library and on the bottom the MS/MS acquired in this study.


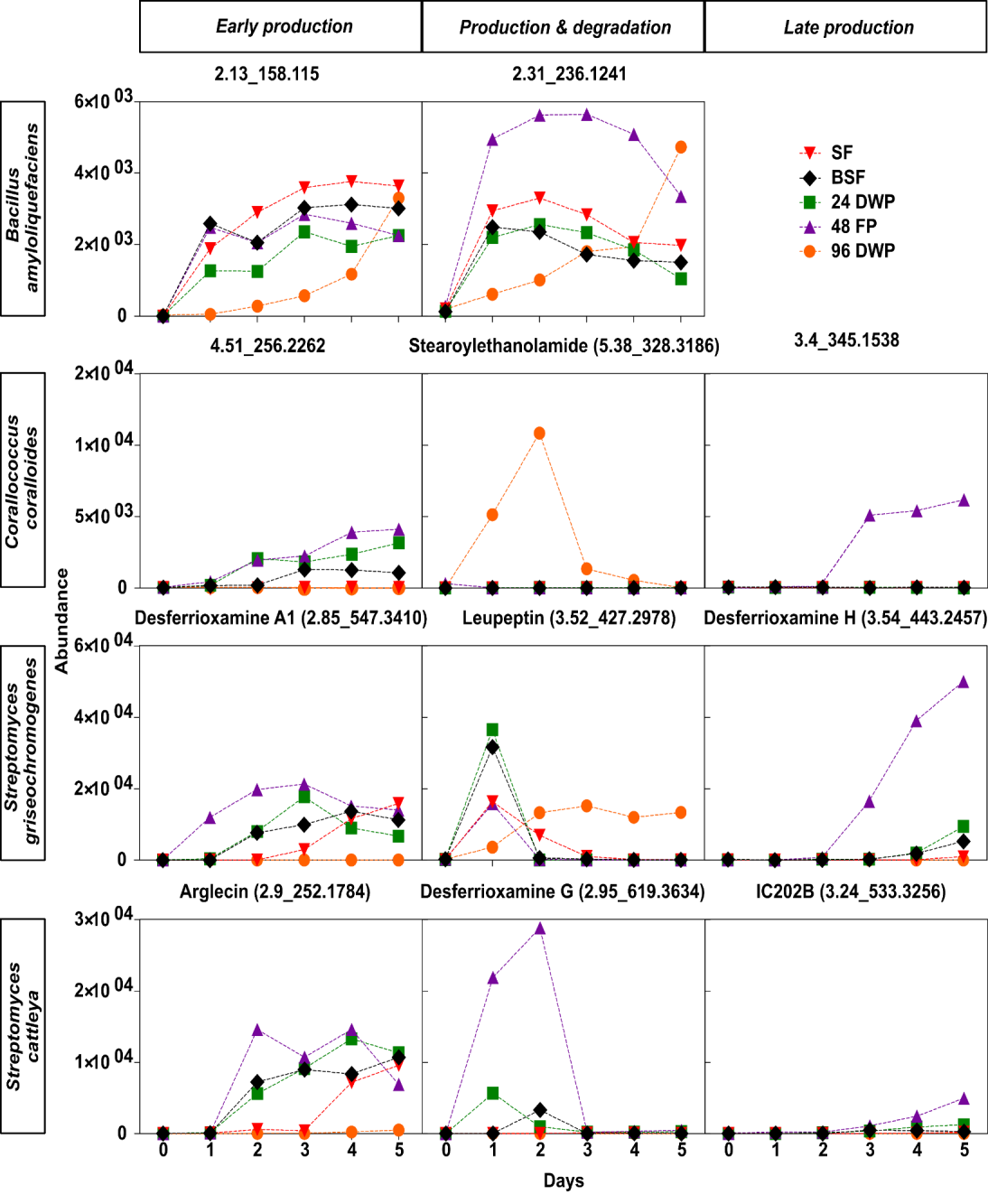


**Figure S3.** Examples of production profiles from MFs produced during cultivation of *B. amyloliquefaciens, C. coralloides, S. griseochromogenes* and *S. cattleya* in SF (red inverted triangle), BSF (black diamonds), 24 DWP (green squares), 48 FP (purple triangles) and 96 DWP (orange circles). MFs that were observed after 1 or 2 days, increasing to a certain intensity until the end of the cultivation, were grouped as *early production* profile. The *production & degradation* profile was assigned to MFs that appeared and disappeared during cultivation. MFs were assigned as *late production* when observed after 3 days or more during the cultivation and showed a continuous increase until the end. MFs were labeled with the name and the feature (retention time_*m/z*).


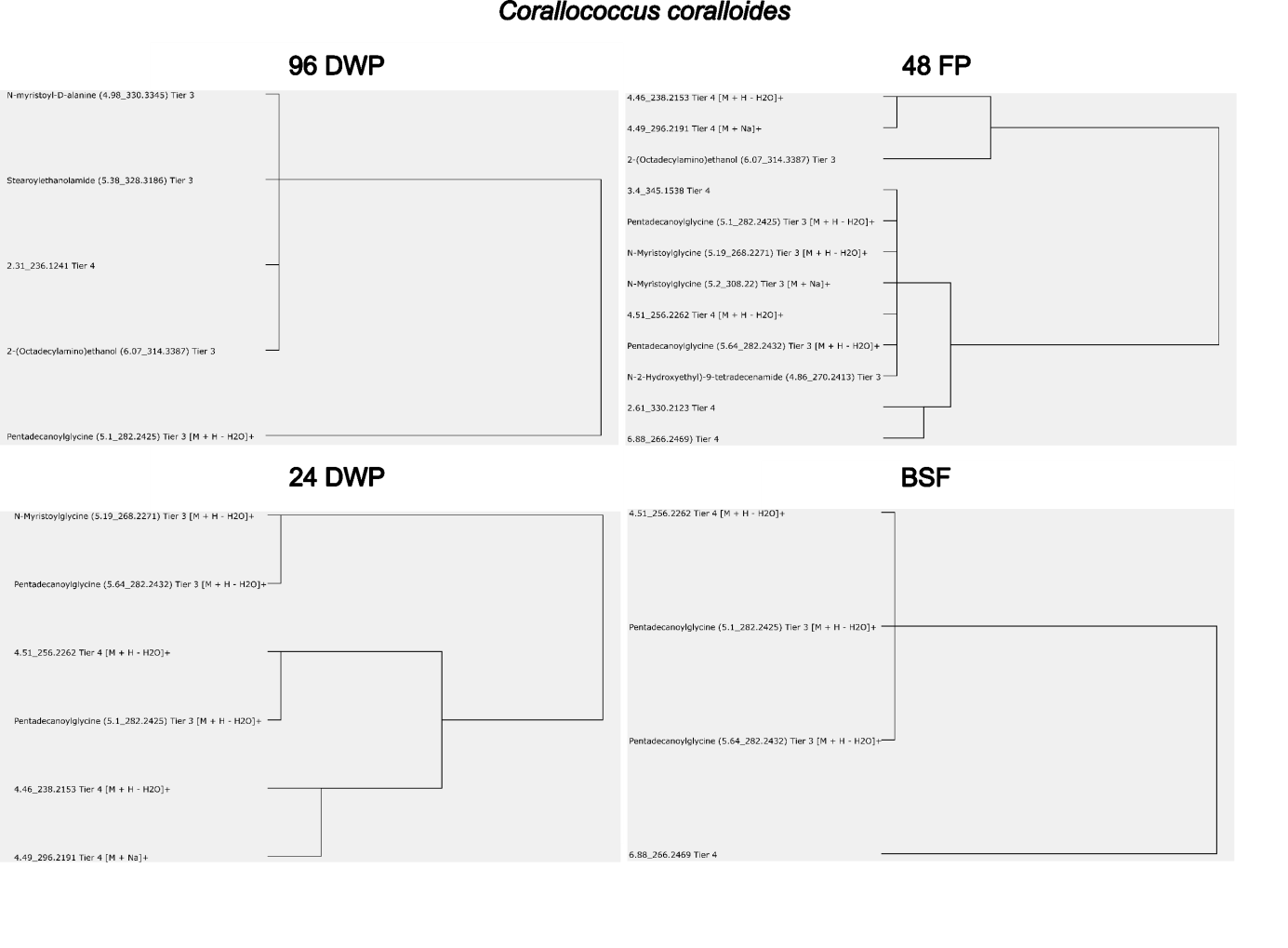


**Figure S4.** Hierarchical clustering analysis from MFs produced during cultivation of *C. coralloides* in BSF, 24 DWP, 48 FP and 96 DWP. The resulting dendrogram displays the clustering of all observed MFs based on their production profiles. Each MF was labelled with a name, feature (retention time_*m/z*) and annotation confidence level (Tier 1 - 4). In-source fragments are marked with the name of the precursor ion and the feature. All m/z represents [M + H] +, unless specified otherwise.


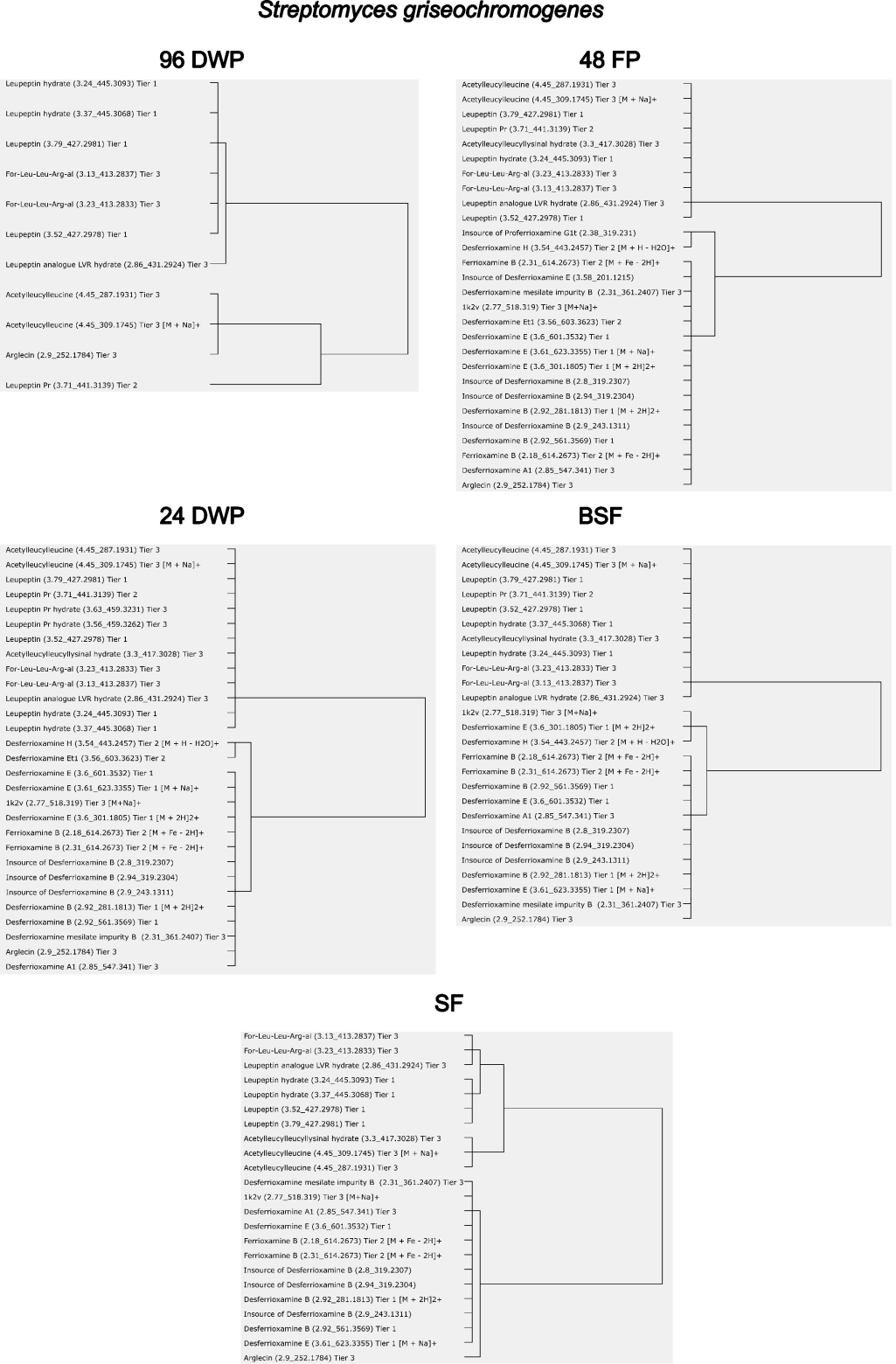


**Figure S5.** Hierarchical clustering analysis from MFs produced during cultivation of *S. griseochromogenes* in SF, BSF, 24 DWP, 48 FP and 96 DWP. The resulting dendrogram displays the clustering of all observed MFs based on their production profiles. Each MF was labelled with a name, feature (retention time_*m/z*) and annotation confidence level (Tier 1 – 4). In-source fragments are marked with the name of the precursor ion and the feature. All m/z represents [M + H] +, unless specified otherwise.


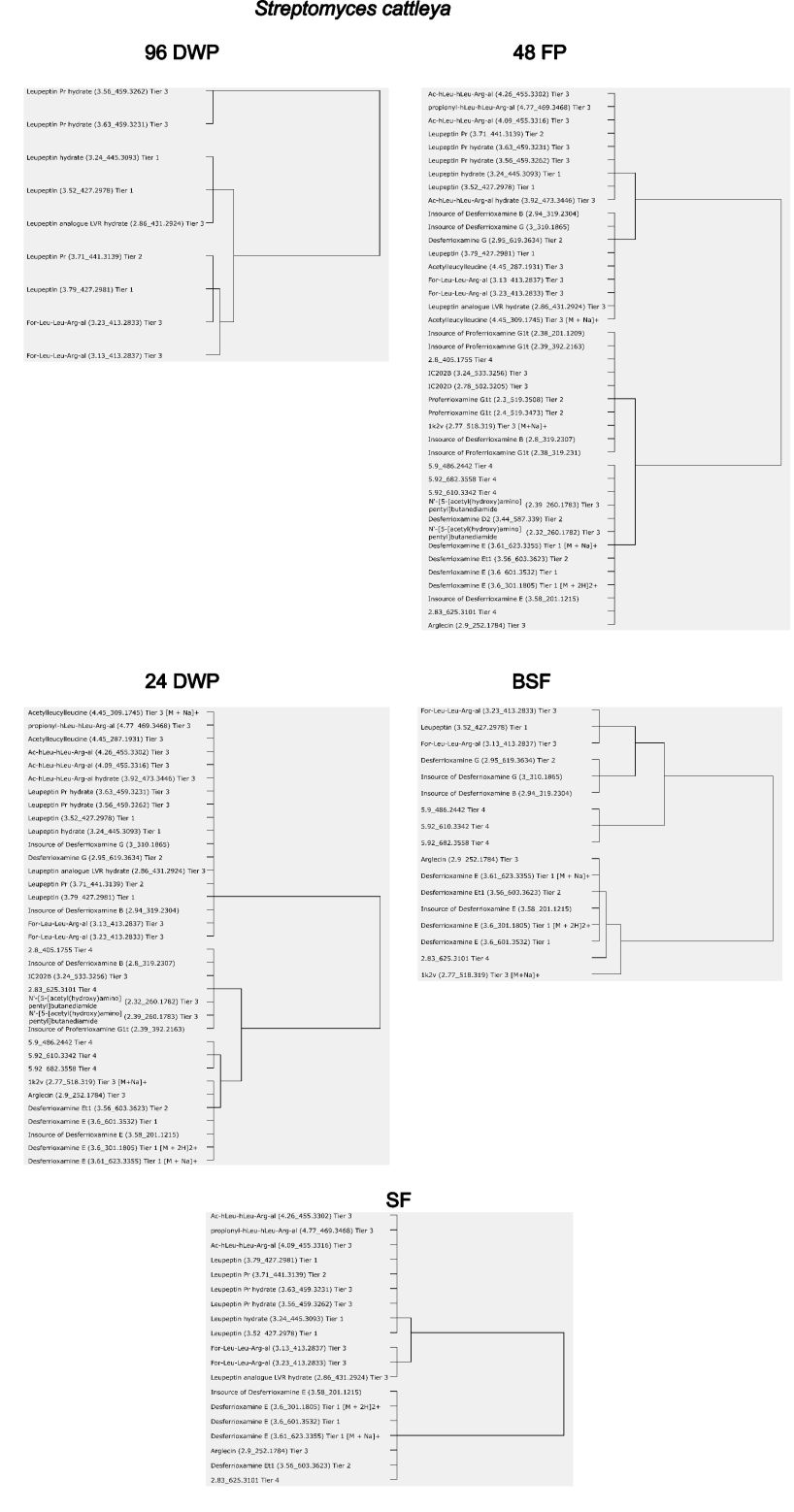


**Figure S6.** Hierarchical clustering analysis from MFs produced during cultivation of *S. cattleya* in SF, BSF, 24 DWP, 48 FP and 96 DWP. The resulting dendrogram displays the clustering of all observed MFs based on their production profiles. Each MF was labelled with a name, feature (retention time_*m/z*) and annotation confidence level (Tier 1 – 4). In-source fragments are marked with the name of the precursor ion and the feature. All m/z represents [M + H] +, unless specified otherwise.**
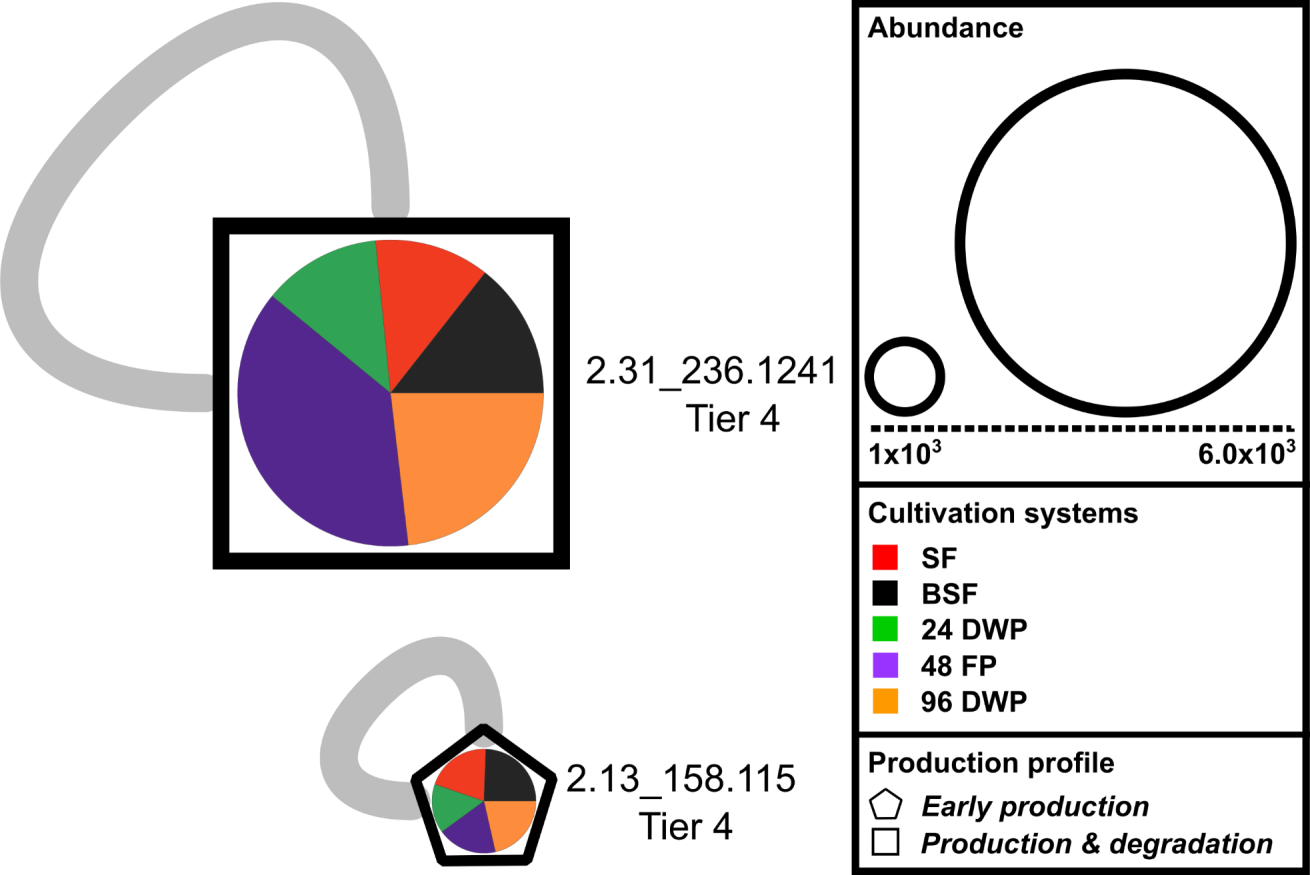
**

**Figure S7.** Molecular network of detected SMs of *B. amyloliquefaciens* in 96 DWP (orange), 24 DWP (green), 48 FP (purple) SF (red) and BSF (black)*.* labeled with feature (retention time_*m/z*) and annotation confidence level. All m/z represents [M + H] +, unless specified otherwise. The appearance of SMs and the highest observed abundance in the different cultivation systems are displayed as a pie chart for each mass. The size of the pie charts represents the highest observed intensity in this study. The shape around the pie chart represents the production profile.


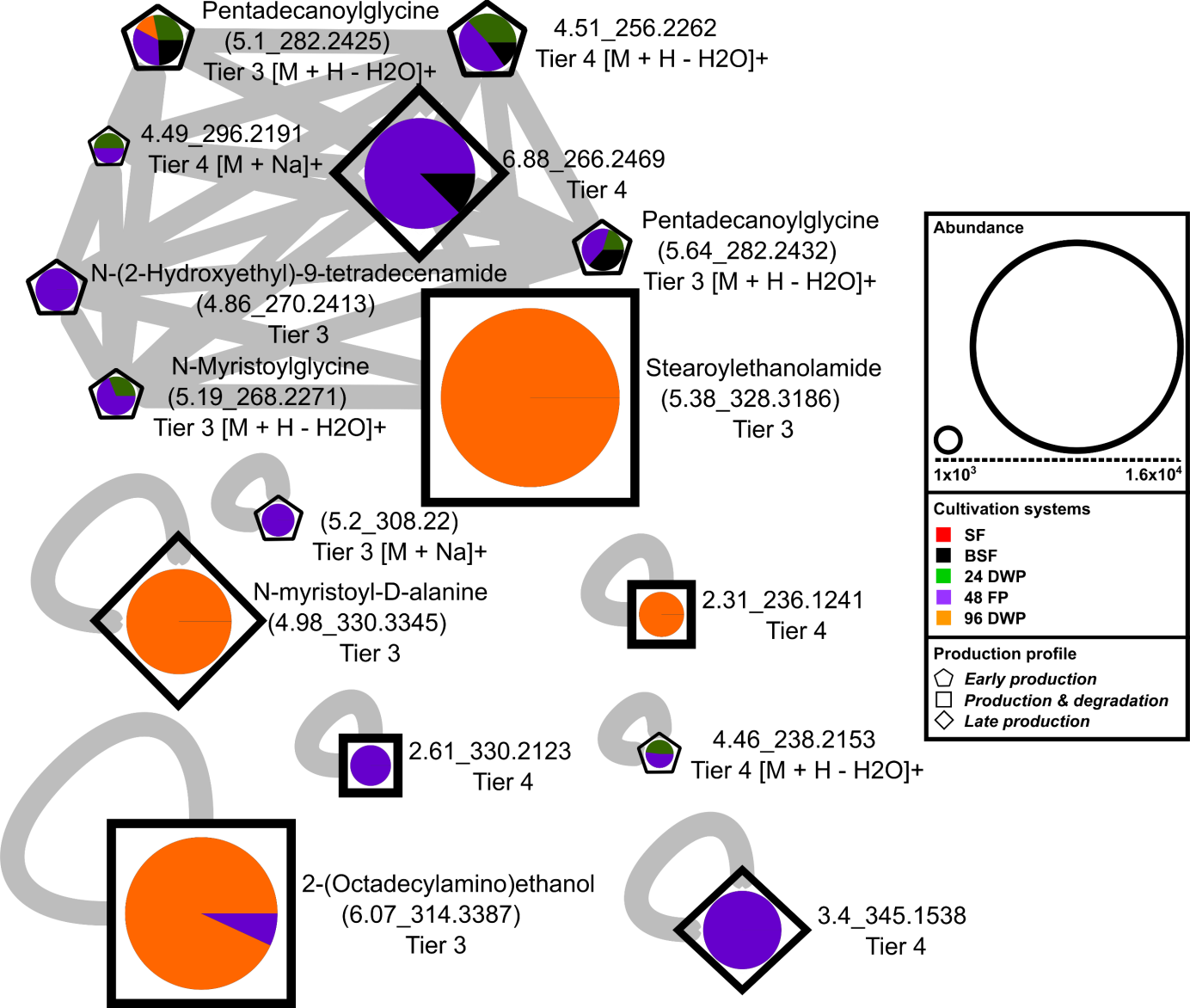


**Figure S8.** Molecular network of detected SMs of *C. coralloides* in 96 DWP (orange), 24 DWP (green), 48 FP (purple) SF (red) and BSF (black). labeled with name, feature (retention time_*m/z*) and annotation confidence level. In-source fragments are marked with name of precursor ion and feature. All m/z represents [M + H] +, unless specified otherwise. The appearance of SMs and the highest observed abundance in the different cultivation systems are displayed as a pie chart for each mass. The size of the pie charts represents the highest observed intensity in this study. The shape around the pie chart represents the production profile.


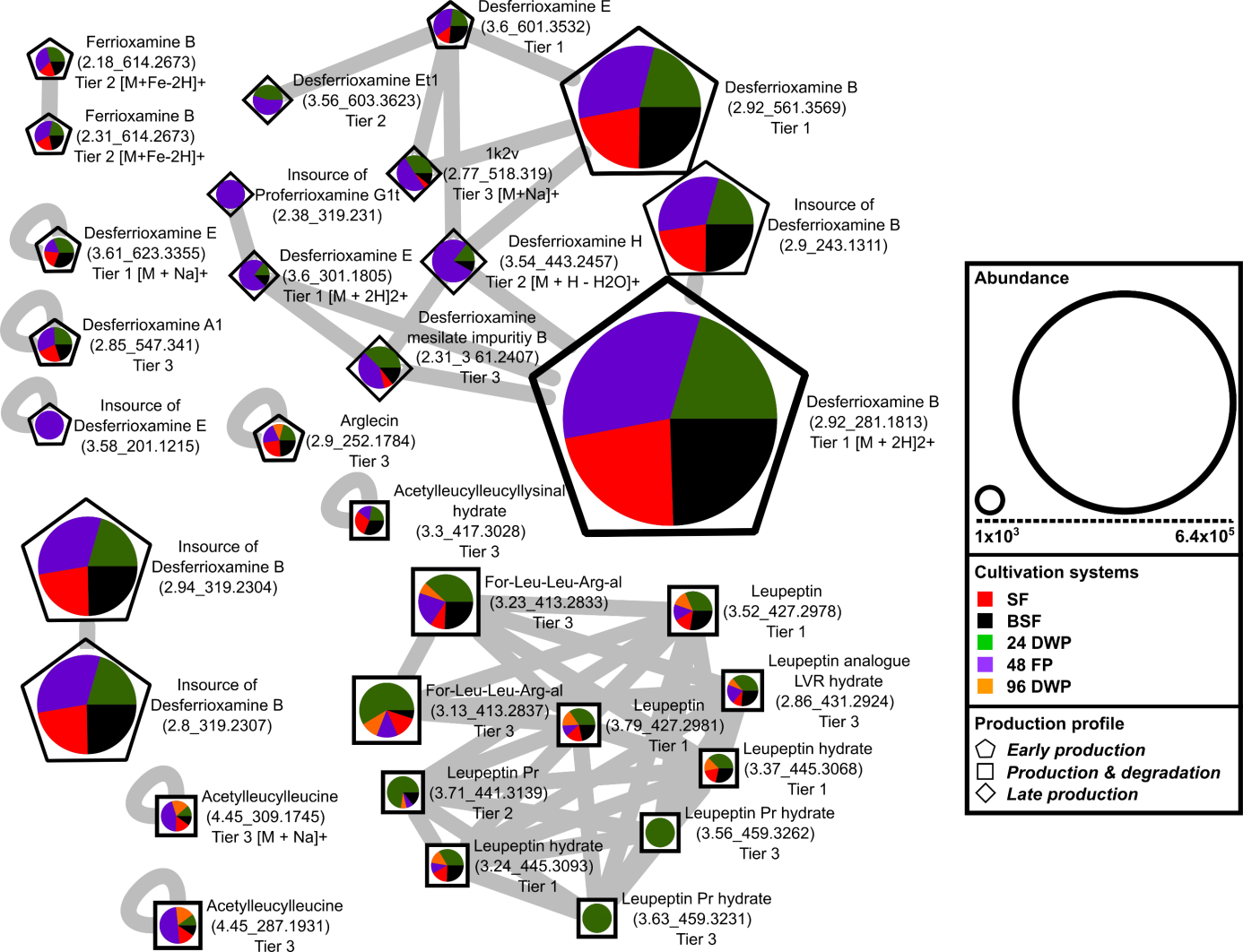


**Figure S9.** Molecular network of detected SMs of *S. griseochromogenes* in 96 DWP (orange), 24 DWP (green), 48 FP (purple) SF (red) and BSF (black). labeled with name, feature (retention time_*m/z*) and annotation confidence level. In-source fragments are marked with name of precursor ion and feature. All m/z represents [M + H] +, unless specified otherwise. The appearance of SMs and the highest observed abundance in the different cultivation systems are displayed as a pie chart for each mass. The size of the pie charts represents the highest observed intensity in this study. The shape around the pie chart represents the production profile.

**Figure S10.** Chemical structure, name and sum formula of detected *N*-Acyl-amino-acids in this study. SEA = Stearoylethanolamide, 2OAE = 2-(Octadecaylamino)ethanol, PDG = Pentadecanoylglycine, MA = N-Myristoyl-D-alanine, MG = N-Myristoylglycine, 2HE9TA = N-(2-hydroxyethyl)-9-tetradecenamide.

**Figure S11.** Chemical structure, name and sum formula of detected Leupeptin structures in this study. PhLhLAA = propionyl -hLeu-hLeu-Arg-al, FLLAA = For-Leu-Leu-Arg-al, AhLhLAA = Ac-hLeu-hLeu-Arg-al, LALVRH = Leupeptin analogue LVR hydrate, AhLhLAAH = Ac-hLeu-hLeu-Arg-al hydrate, ALLLH = Acetylleucylleucyllysinal hydrate, LP Pr = Leupeptin Pr, LPPrH = Leupeptin Pr hydrate, ALL = Acetylleucylleucine, LP - Leupeptin, LPH = Leupeptin hydrate, AL = Arglecin.


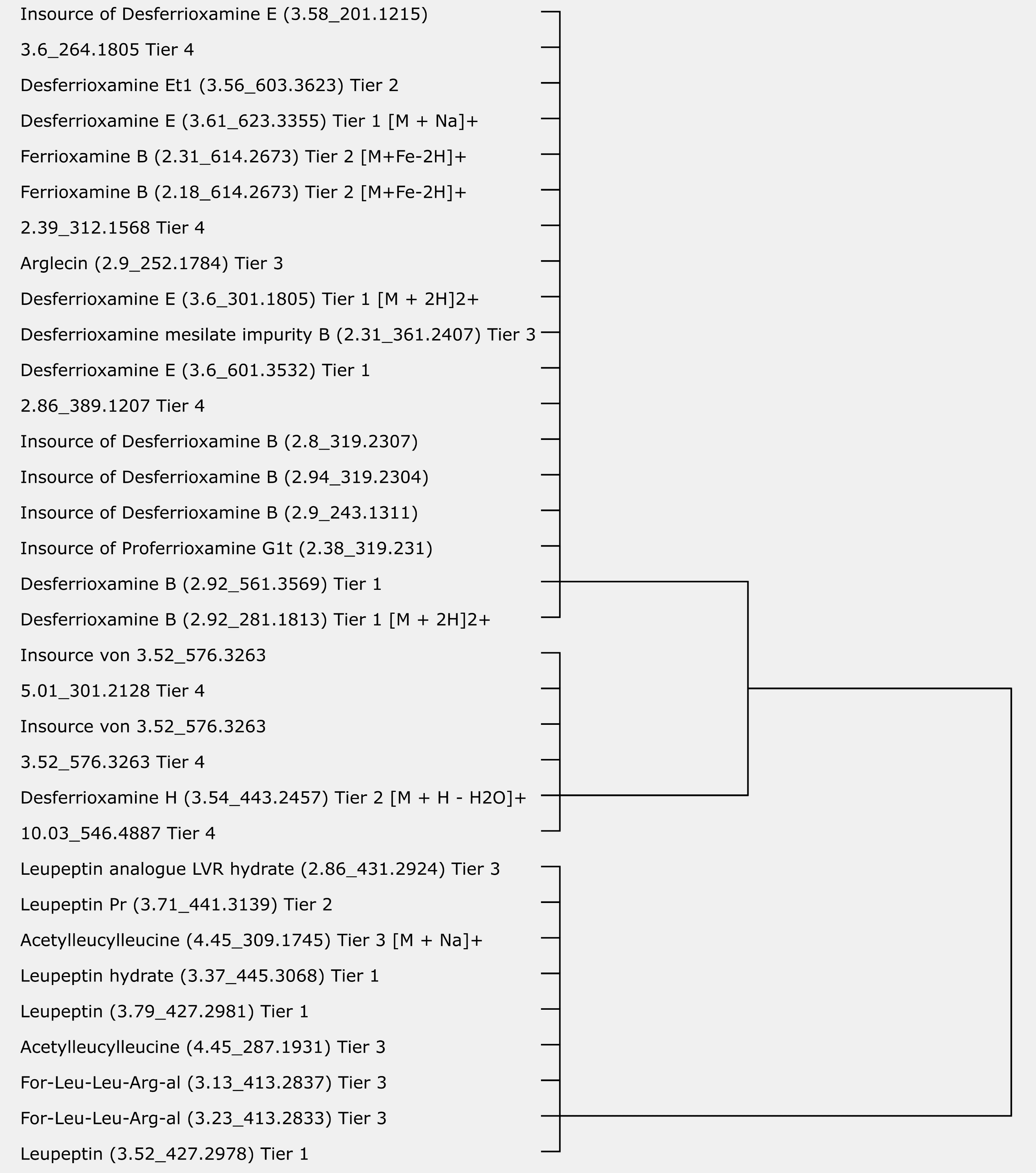


**Figure S12.** Hierarchical clustering analysis from MFs produced during cultivation of *S. griseochromogenes* in STR. The resulting dendrogram displays the clustering of all observed MFs based on their production profiles. Each MF was labelled with a name, feature (retention time_*m/z*) and annotation confidence level. In source fragments are marked with the name of the precursor ion and the feature. All m/z represents [M + H] +, unless specified otherwise.


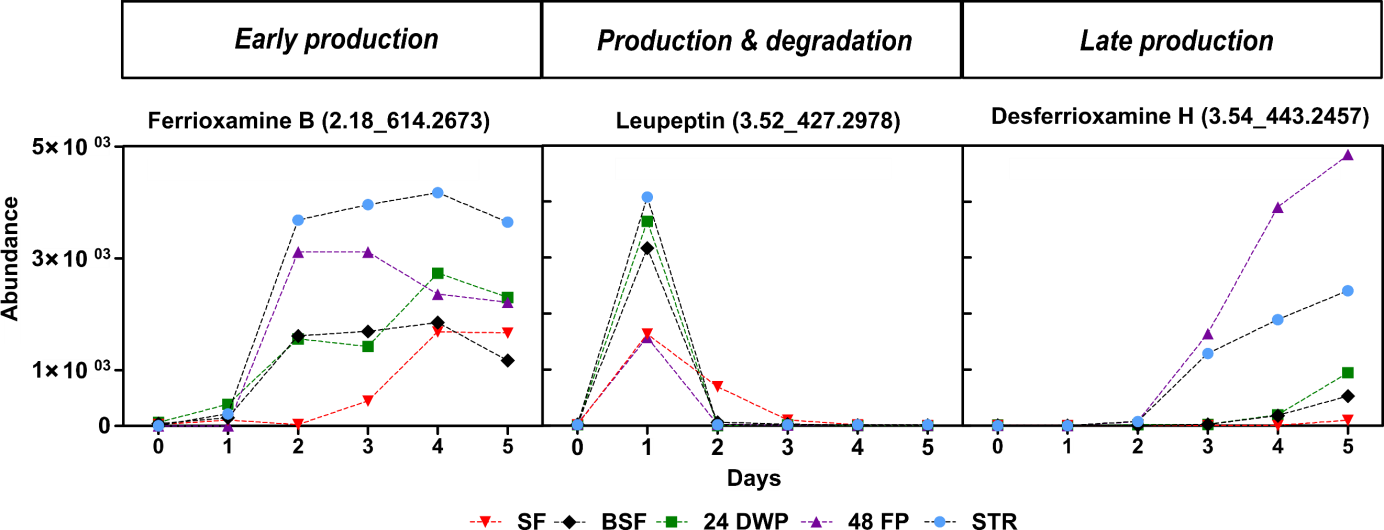


**Figure S13.** Examples of production profiles from MFs produced during cultivation of *S. griseochromogenes* in SF (red inverted triangle), BSF (black diamonds), 24 DWP (green squares), 48 FP (purple triangles) and STR (blue circles). MFs that were observed after 1 or 2 days, increasing to a certain intensity until the end of the cultivation, were grouped as *early production* profile. The *production & degradation* profile was assigned to MFs that appeared and disappeared during cultivation. MFs were assigned as *late production* when observed after 3 days or more during the cultivation and showed a continuous increase until the end. MFs were labeled with the name and the feature (retention time_*m/z*).
